# A role for activated Cdc42 in glioblastoma multiforme invasion

**DOI:** 10.18632/oncotarget.10925

**Published:** 2016-07-29

**Authors:** Hidehiro Okura, Brian J. Golbourn, Uswa Shahzad, Sameer Agnihotri, Nesrin Sabha, Jonathan R. Krieger, Carlyn A. Figueiredo, Alan Chalil, Natalie Landon-Brace, Alexandra Riemenschneider, Hajime Arai, Christian A. Smith, Songli Xu, Stefan Kaluz, Adam I. Marcus, Erwin G. Van Meir, James T. Rutka

**Affiliations:** ^1^ The Arthur and Sonia Labatt Brain Tumour Research Centre, The Hospital for Sick Children, Peter Gilgan Centre for Research and Learning, Toronto, Ontario, M5G 0A4, Canada; ^2^ Department of Neurosurgery, Juntendo University School of Medicine, Bunkyo-ku, Tokyo, Japan; ^3^ Department of Surgery, University of Toronto, Toronto, Ontario, M5T 1P5, Canada; ^4^ Department of Neurosurgery, Emory University School of Medicine, Atlanta, Georgia, 30307, USA; ^5^ Department of Hematology and Medical Oncology, Emory University School of Medicine, Atlanta Georgia, 30307, USA; ^6^ Winship Cancer Institute, Emory University School of Medicine, Atlanta, Georgia, 30307, USA; ^7^ Genetics and Genome Biology, Hospital for Sick Children Research Institute, Toronto, Ontario, M5G 0A4, Canada; ^8^ SPARC Biocentre, The Hospital for Sick Children, Toronto, Ontario, M5G 0A4, Canada

**Keywords:** cdc42, glioblastoma, migration, invasion, IQGAP1 and pFAK

## Abstract

Cdc42 is a Rho-GTPase which plays a major role in regulating cell polarity and migration by specifying the localization of filopodia. However, the role of Cdc42 in GBM invasion has not been thoroughly investigated. We generated stable doxycycline-inducible clones expressing wild type (WT)-, constitutively active (CA)-, and dominant negative (DN)-Cdc42 in three different human glioma cell lines. Expression of CA-Cdc42 significantly increased the migration and invasive properties of malignant glioma cells compared to WT and DN-Cdc42 cell clones, and this was accompanied by a greater number of filopodia and focal adhesion structures which co-localize with phosphorylated focal adhesion kinase (FAK). By mass spectrometry and immunoprecipitation studies, we demonstrated that activated Cdc42 binds to IQGAP1. When implanted orthotopically in mice, the CA-Cdc42 expressing glioma cells exhibited enhanced local migration and invasion, and led to larger tumors, which significantly reduced survival. Using the Cancer Genome Atlas dataset, we determined that high Cdc42 expression is associated with poorer progression free survival, and that Cdc42 expression is highest in the proneural and neural subgroups of GBM. In summary, our studies demonstrate that activated Cdc42 is a critical determinant of the migratory and invasive phenotype of malignant gliomas, and that its effect may be mediated, at least in part, through its interaction with IQGAP1 and phosphorylated FAK.

## INTRODUCTION

GBM is characterized by uncontrolled tumor cell proliferation, diffuse infiltration into surrounding normal brain tissue, and impairment of neurological function. Unremitting growth and invasion of GBM into critical areas of the brain make complete neurosurgical extirpation extremely difficult, and lead to recurrence and subsequent early mortality [1]. Accordingly, investigations aimed at increasing our understanding of the molecular mechanisms of brain invasion by GBM are essential to develop future therapeutic strategies.

Cell movement depends on the reorganization of the actin cytoskeleton and related cytoskeletal proteins with directed protrusions at the front of the cell and retraction at the rear. There is increasing evidence that the small cytoskeletal Rho-GTPases play a primary role in migration and invasion of GBM cells [2–5]. They regulate complex molecular events by cycling between an inactive GDP-bound and active GTP-bound forms [6]. Binding to GTP is prompted by Rho guanine nucleotide exchange factors (GEFs), and GTP hydrolysis is catalyzed by Rho-GTPase activating proteins (GAPs) [7]. In their active GTP-bound form, Rho-GTPases are able to interact with numerous effector molecules to initiate downstream responses until GTP hydrolysis returns them into their inactive state [1, 6].

The three best characterized and studied members of the Rho-GTPases are Rho, Rac, and Cdc42 [6, 8], and early studies showed they control cell morphology and actin cytoskeletal reorganization [7, 9]. Subsequent studies broadened their functions into the regulation of focal adhesion formation, membrane ruffling, filopodia formation, vesicular trafficking, cell proliferation, cell polarity, gene expression, and cell stemness in conjunction with their regulators and downstream effectors [7, 10–13]. Finally, there have been reports that show that aberrant activation of Rho-GTPases contributes to tumorigenesis [7, 14, 15].

We have previously studied the roles of Rho and Rac in glioma cytoskeletal reorganization and migration [3, 4, 16]. In addition, we have shown that GBM cells demonstrate amoeboid migration through Rho-associated protein kinase activation [5]. However, unlike the situation for Rho and Rac, little is known about the role of Cdc42 in human brain tumors including GBM. Accordingly, we examined the role of activated Cdc42 in GBM cells especially with respect to their tumor cell invasion and migration properties. Our studies demonstrate an interaction of activated Cdc42 with IQGAP1 and phosphorylated focal adhesion kinase (FAK) – an interaction which may contribute to the increased aggressiveness of and invasiveness of malignant gliomas.

## RESULTS

### Cdc42 knockdown by Cdc42-specific siRNA reduces migration, invasion and alters glioma cell morphology

**Figure 1:**
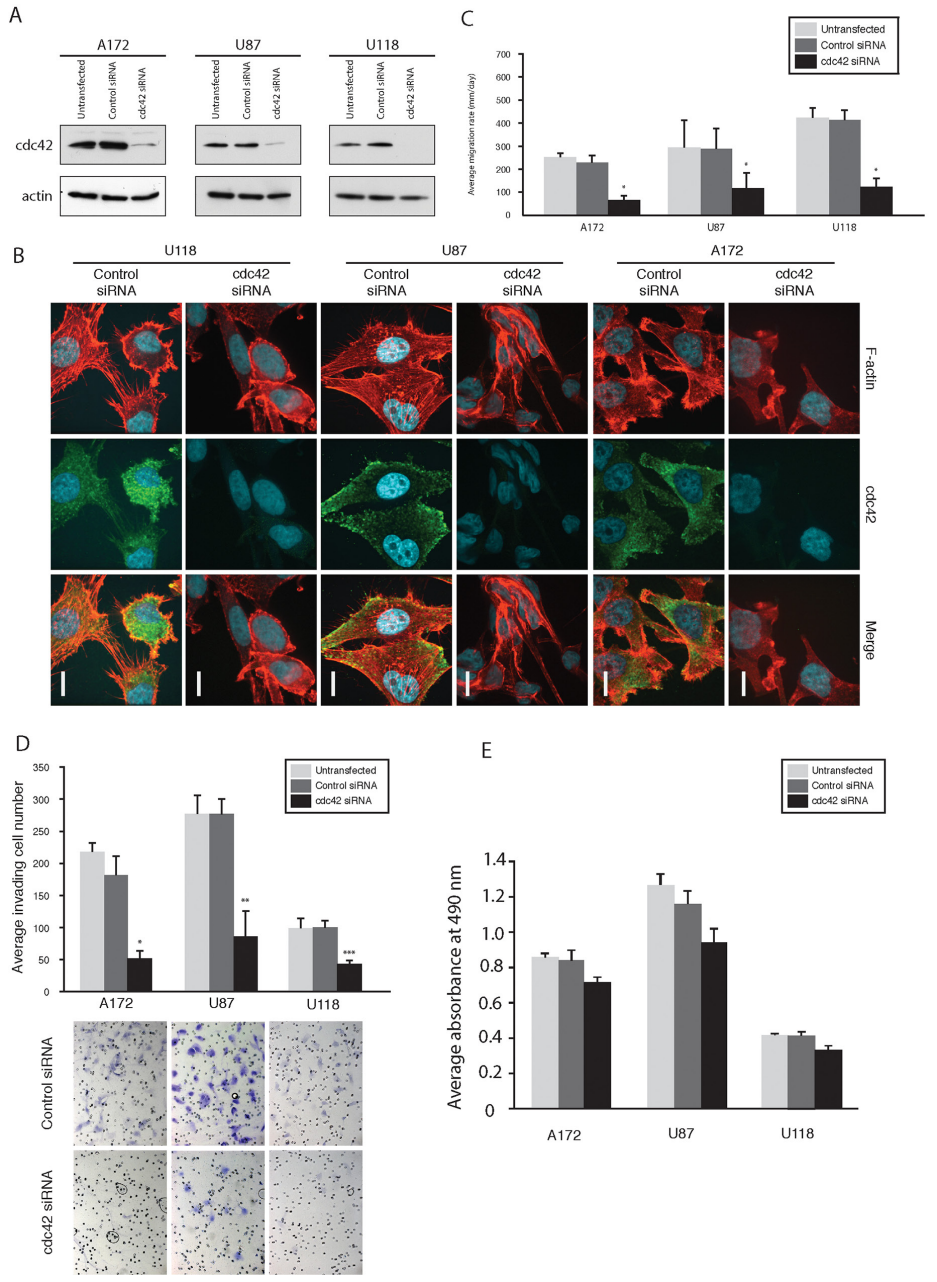
Knockdown of Cdc42 modulates morphology, suppress migration, and invasion of GBM cells **A.** Knockdown of Cdc42 by siRNA in A172, U87MG, and U118MG cells. **B.** Morphological alterations of A172, U87MG and U118MG cells after knocking down Cdc42 siRNA. The cells were labeled with F-actin and immunostained with anti-Cdc42 antibody. Scale Bar, 15 μm. **C.** Results of cell migration assays. Bar graphs show the average migration rate calculated as the change in the diameter of the circle circumscribing the cell population over a 24 hrs period. (*p < 0.05). **D.** Results of cell invasion assays. Bar graph showing the average invading cell number within 24 hrs (*P = 0.003, **P = 0.016, ***P = 0.013). magnification x200. **E.** MTS assay for viability of cells following Cdc42 knockdown. The average absorbance at 490 nm after the incubation with MTS.

A significant decrease in Cdc42 expression was demonstrated by western blot analysis in A172, U87MG and U118MG human glioma cells at 72 hrs post-transfection (Figure 1A). The effects of modulation of Cdc42 depletion on cell morphology were subsequently examined by immunofluorescence staining for Cdc42 and actin filaments (Figure 1B). Cdc42 localizes to the cytoplasm and is found in particular at the leading edge of cells where modulation of filopodia formation as well as actin organization occur [6]. As expected, we found that Cdc42 co-localized with filopodia in glioma cells, and Cdc42 knockdown with siRNA decreased filopodia formation and induced a change in shape towards polygonal (A172 and U87MG), or more rounded (U118MG) morphologies, accompanied by a reduction in actin stress fibers (Figure 1B). All cell lines demonstrated significantly reduced migration rates following knockdown of Cdc42 compared to control siRNA in a radial cell migration assay (*p*<0.05, Figure 1C). Furthermore, Cdc42 knockdown significantly decreased the invasiveness of A172 (*p*=0.003), U87MG (*p*=0.016), and U118MG cells (*p*=0.013) in a matrigel invasion assay as compared to control siRNA (Figure 1D). Finally, we examined glioma cell viability after Cdc42 specific siRNA knockdown. There was a slight decrease in Cdc42 expression after siRNA knockdown, but this was not statistically significant (Figure 1E). Taken together, these results suggest that knockdown of Cdc42 reduces filopodia formation and actin stress fiber formation in glioma cells, and decreases their migratory and invasive phenotype, whereas there is little impact on cell viability.

### Effects of inducible overexpression of Cdc42 on glioma cell migration and invasion

**Figure 2:**
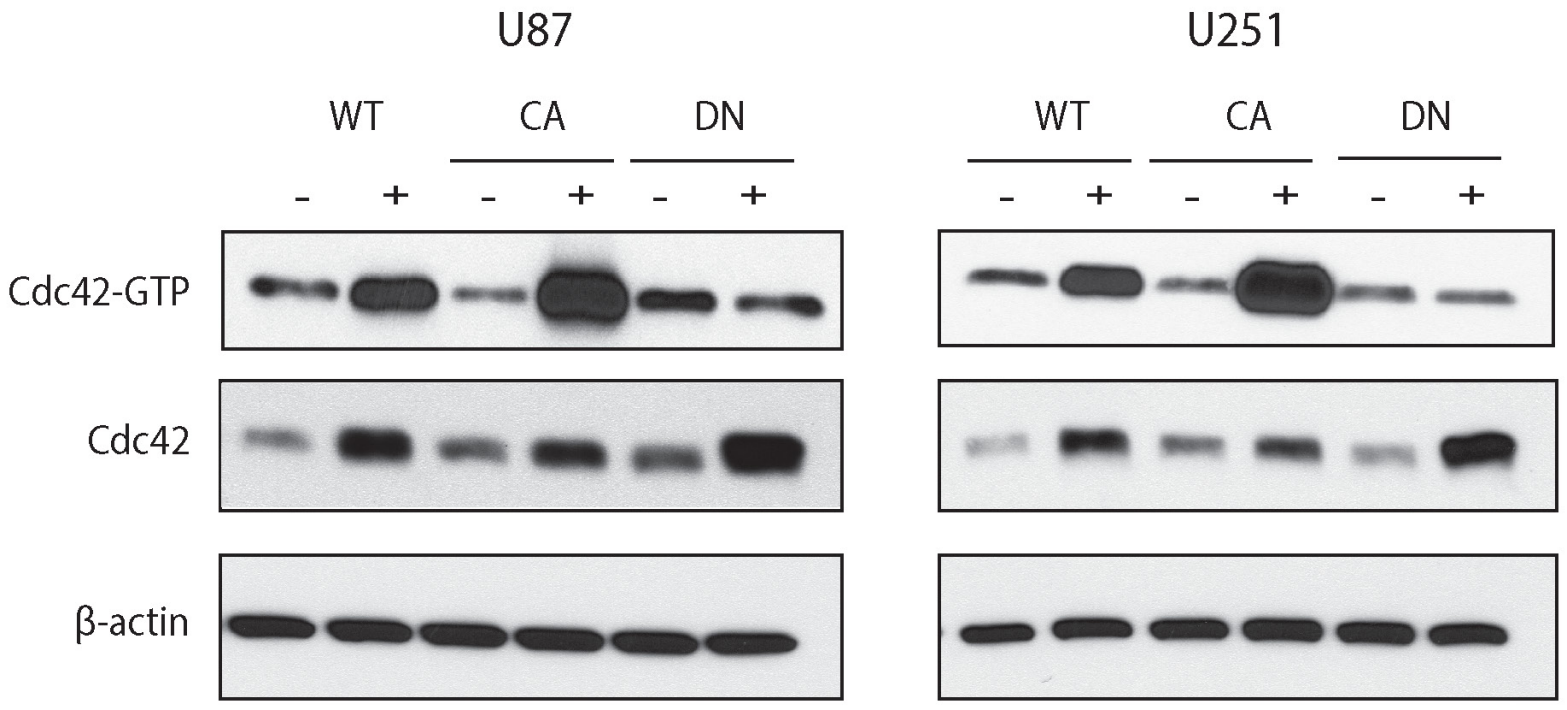
Doxycycline inducible cell lines expressing WT-, CA-, and DN-Cdc42 in U87MG and U251MG The total amount of Cdc42 is increased in the presence of doxycycline in all cell lines. Activated Cdc42 (Cdc42-GTP) is increased in WT- and is even higher in CA-Cdc42 in the presence of doxycycline. However, Cdc42-GTP in DN-Cdc42 cells treated with doxycycline has the same expression level as doxycycline negative cultures despite an increased expression of total amount of Cdc42.

Conversely, to investigate the role of Cdc42 overexpression in glioma cells, we generated stable glioma cell lines with doxycycline-inducible wild type (WT), constitutively active (CA), and dominant negative (DN) Cdc42. To confirm that overexpression of Cdc42 resulted in augmented activity, we used a Cdc42 activation assay using each cell U87MG- and U251MG-Cdc42 clones. Following the administration of doxycycline for 72 hrs, overexpressed WT- or CA-Cdc42 were GTP bound, while the DN-Cdc42 was inactive (Figure 2, top row). Each clone showed maximal expression of total Cdc42 after 72 hrs induction relative to uninduced controls (Figure 2, middle row).

### Effects of Cdc42 activation levels on glioma proliferation

**Figure 3:**
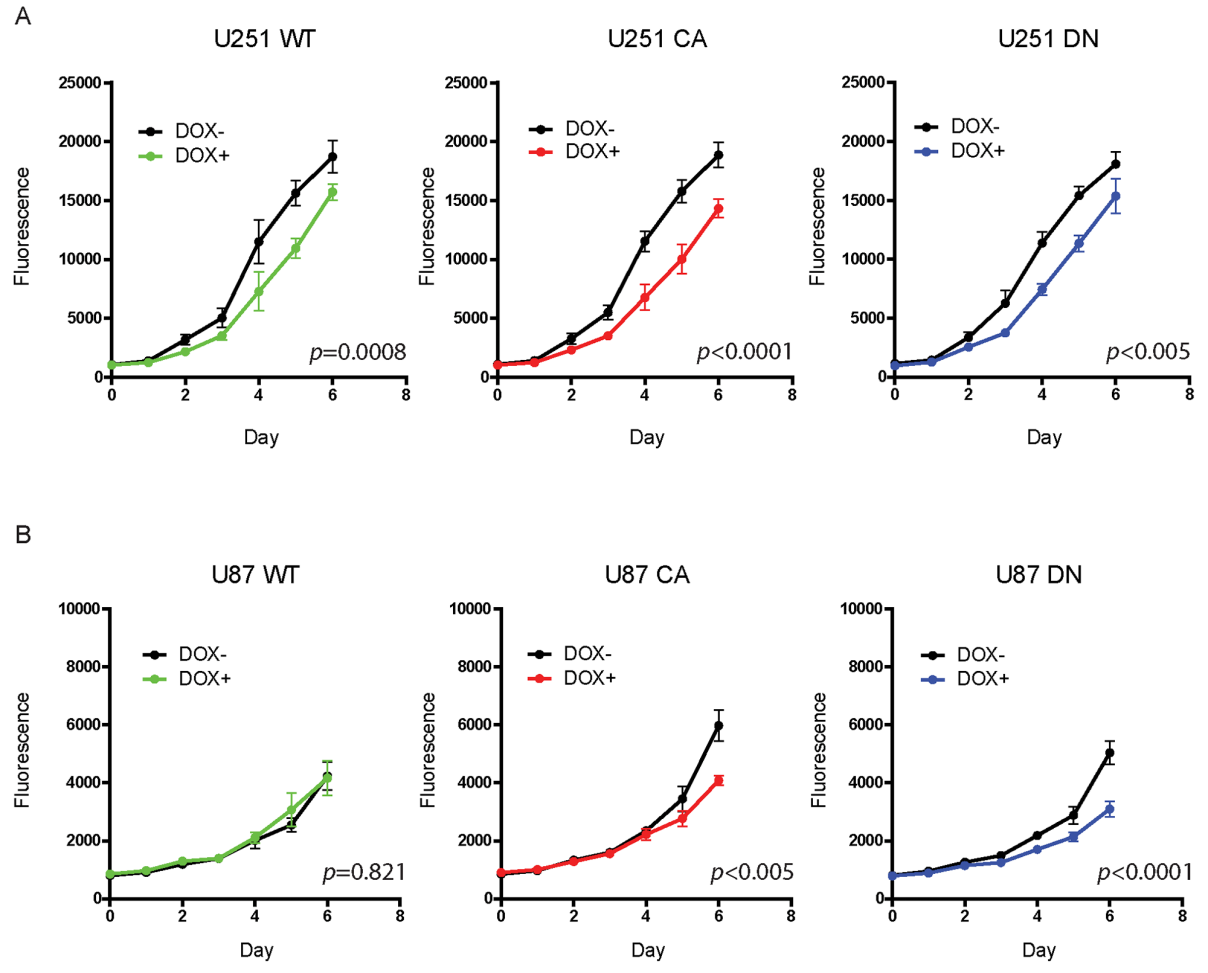
Doxycycline treatment to induce Cdc42 does not increase proliferation **A.** All three U251 MG cell clones demonstrated a similar decrease in cell proliferation by day six. **B.** The U87MG inducible cells also demonstrated a similar decrease in cell proliferation in CA- and DN-cell clones but not in WT.

The effect of overexpression of activated Cdc42 on cell proliferation was assessed by Alamar Blue staining. Before cell seeding, Cdc42 expression was induced in U251MG and U87MG with doxycycline for 72 hrs, and the cells kept in doxycycline for the duration of the experiment. All three U251MG cell clones demonstrated a significant and somewhat similar decrease in cell proliferation (WT; *p*=0.0008, CA; *p*<0.0001, DN; *p*<0.005) on day 6 (Figure 3A). The U87MG inducible cells also demonstrated a significant and similar decrease in cell proliferation (CA; *p*<0.005, DN; *p*<0.0001), with the exception of the WT cell clone (*p*=0.821) (Figure 3B). These results suggest that Cdc42 activation levels *per se* do not influence glioma proliferation when compared to the results from the other cell clones.

### Activated Cdc42 increases glioma cell migration and invasion

**Figure 4:**
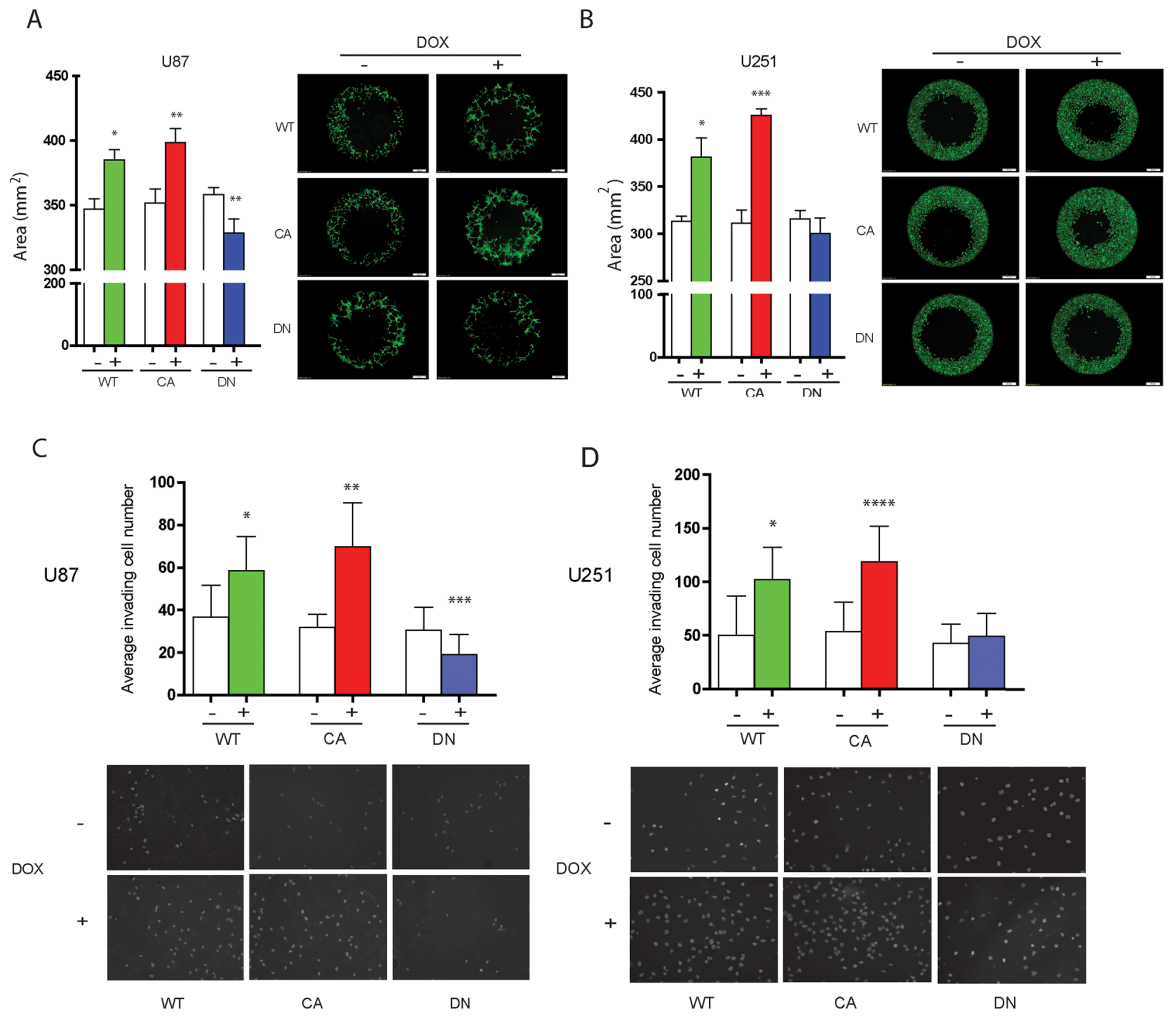
Induced aberrant cdc42 activity alters cell migration and invasion in U87MG and U251MG glioma cells **A, B.** Migration assay of Cdc42 expressing inducible U87MG (A) and U251MG (B) cells after 72 hrs of treatment with or without (+ or −) doxycycline. WT- and CA-Cdc42 expressing cell clones significantly increase migration of U87MG and U251MG cells. DN-Cdc42 significantly reduces migration of U87MG cells but does not change migration potential of U251MG cells (*p* = 0.21). *p < 0.05, **p < 0.01, ***p <0.005. **C, D.** Matrigel invasion assay of Cdc42 expressing inducible U87MG (C) or U251MG (D) cells after 72 hr of treatment with or without (+ or −) doxycycline. WT- and CA-Cdc42 expressing cell clones treated with doxycycline significantly increase invasion of U87MG and U251MG cells. DN-Cdc42 expression reduces invasion of U87MG cells. DN-Cdc42 expression does not change the invasiveness of U251MG cells however (*p* = 0.504). *p < 0.01, **p < 0.0001, ***p < 0.05, ****p < 0.0005.

The Oris^TM^ Cell Migration Assay was used to determine the distance glioma cells migrated at 24 hrs when cell number or viability is not altered by Cdc42 expression levels. WT- and CA-Cdc42 expressing U87MG and U251MG cells significantly increased their migration compared to controls (U87MG WT; *p* < 0.05, CA; *p* < 0.01, U251MG WT; *p* < 0.05, CA *p* < 0.005) (Figure 4A and 4B). In contrast, the migration of DN-Cdc42 expressing cells treated with doxycycline was significantly inhibited in U87MG (*p* < 0.01). Taken together, these results indicated that activated Cdc42 leads to a significant increase in glioma cell migration.

WT- and CA-Cdc42 expressing U87MG and U251MG cells treated with doxycycline exhibited a significant increase in invasion compared to control cells (U87MG WT; *p* < 0.01, CA; *p* < 0.0001, U251MG WT; *p* < 0.01, CA; *p* < 0.0005) (Figure 4C and 4D). The effect was more pronounced in CA-Cdc42 expressing cells, while the U87MG DN-Cdc42 cells showed a significant reduction in cell invasion (*p* < 0.05) (Figure 4C).

### Active Cdc42 enhances the invasion and restores the activity of glioma cells in 3D spheroid cultures

**Figure 5:**
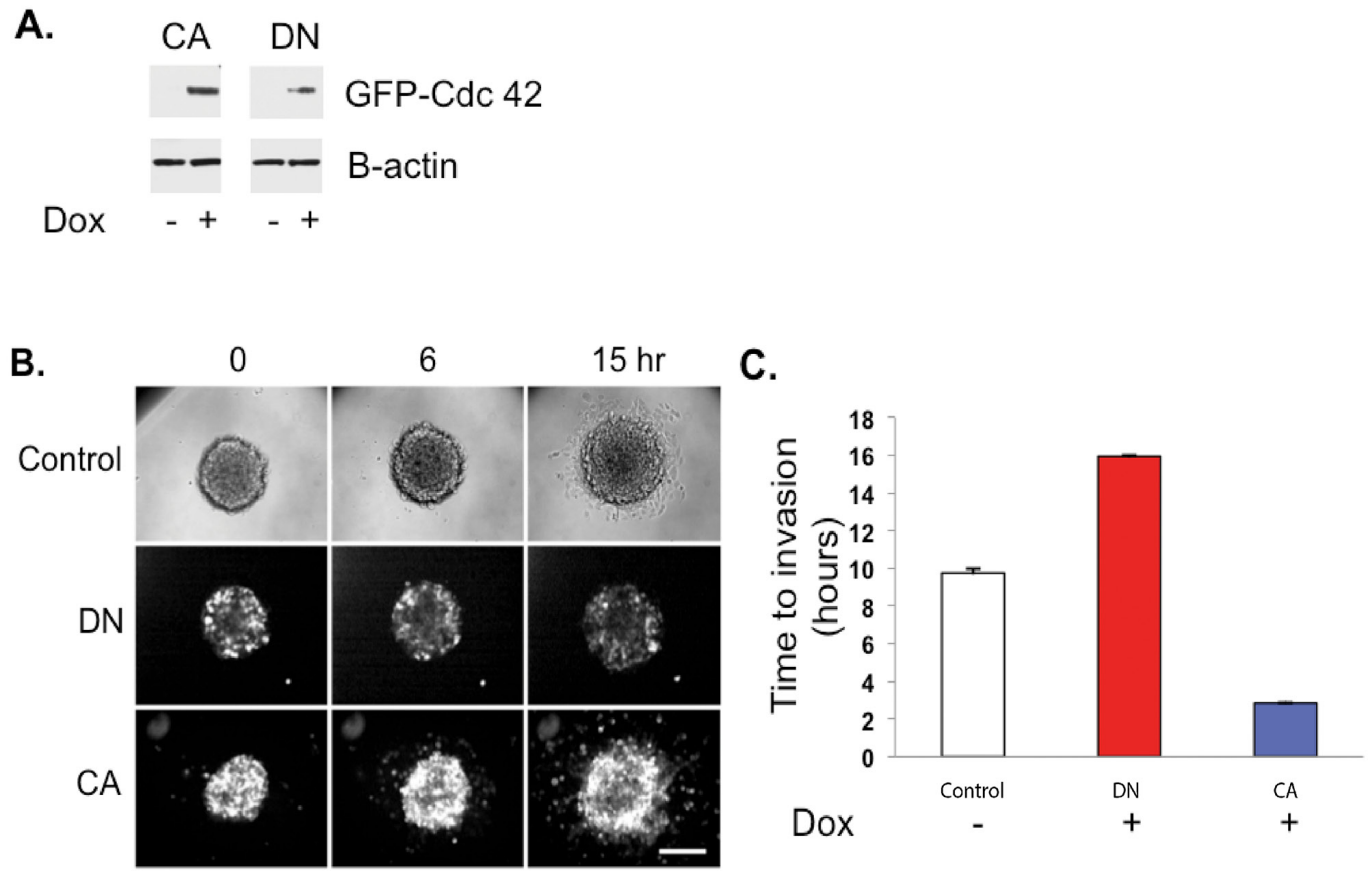
CA-Cdc42 increases three-dimensional spheroid invasion **A.** CA- and DN-Cdc42 expression induced by doxycycline treatment. **B.** Real time imaging of cellular invasion of spheroids embedded in Matrigel was performed using fluorescence imaging. **C.** Quantitative analysis of the 3-D spheroid invasion assay.

LN229 cells were stably transfected with either CA- or DN-Cdc42 mutants, both under doxycycline-inducible promoters (Figure 5A). Intriguingly, 2D proliferation assays demonstrated that CA-Cdc42 expressing LN229 glioma cells increase their viability with doxycycline treatment (data not shown). Real time imaging of cellular invasion of spheroids embedded in Matrigel was performed using fluorescence imaging on a spinning disc confocal in a temperature and CO_2_ controlled environment (Figure 5B, Supplementary Movie S1). Uninduced control spheroids from either clone did not have GFP fluorescence as monitored by brightfield imaging (Figure 5B, Supplementary Movie S1A) and their time to initial invasion was approximately 10 hrs post-embedding into the Matrigel (Figure 5C, Supplementary Movie S1A). In contrast, when active GFP:CA-Cdc42 was induced, cells rapidly invaded after 3.7 hrs (Figure 5C, Supplementary Movie S1B). Doxycycline treatment of untransfected parental cells had no effect on time to invasion (data not shown). Importantly, doxycycline induction in the GFP:DN-Cdc42 spheroids resulted in few cells invading around the 16 hrs time point (Figure 5C, Supplementary Movie S1C), showing that expression of inactive Cdc42 represses invasion. We then analyzed the cell viability of CA-Cdc42 LN229 cells in 3D spheroid cultures. We observed that CA-Cdc42 expression does not increase cell proliferation, suggesting that the significant increase in glioma migration with doxycycline induction is not accompanied by changes in proliferation status (Supplementary Figure S1). Taken together, these data show that inducible activation of Cdc42 generates highly invasive and viable cells within a spheroid model, and inactivation of Cdc42 inhibits invasion.

### Activated Cdc42 decreases and inactive Cdc42 increases survival of glioma-bearing mice

**Figure 6:**
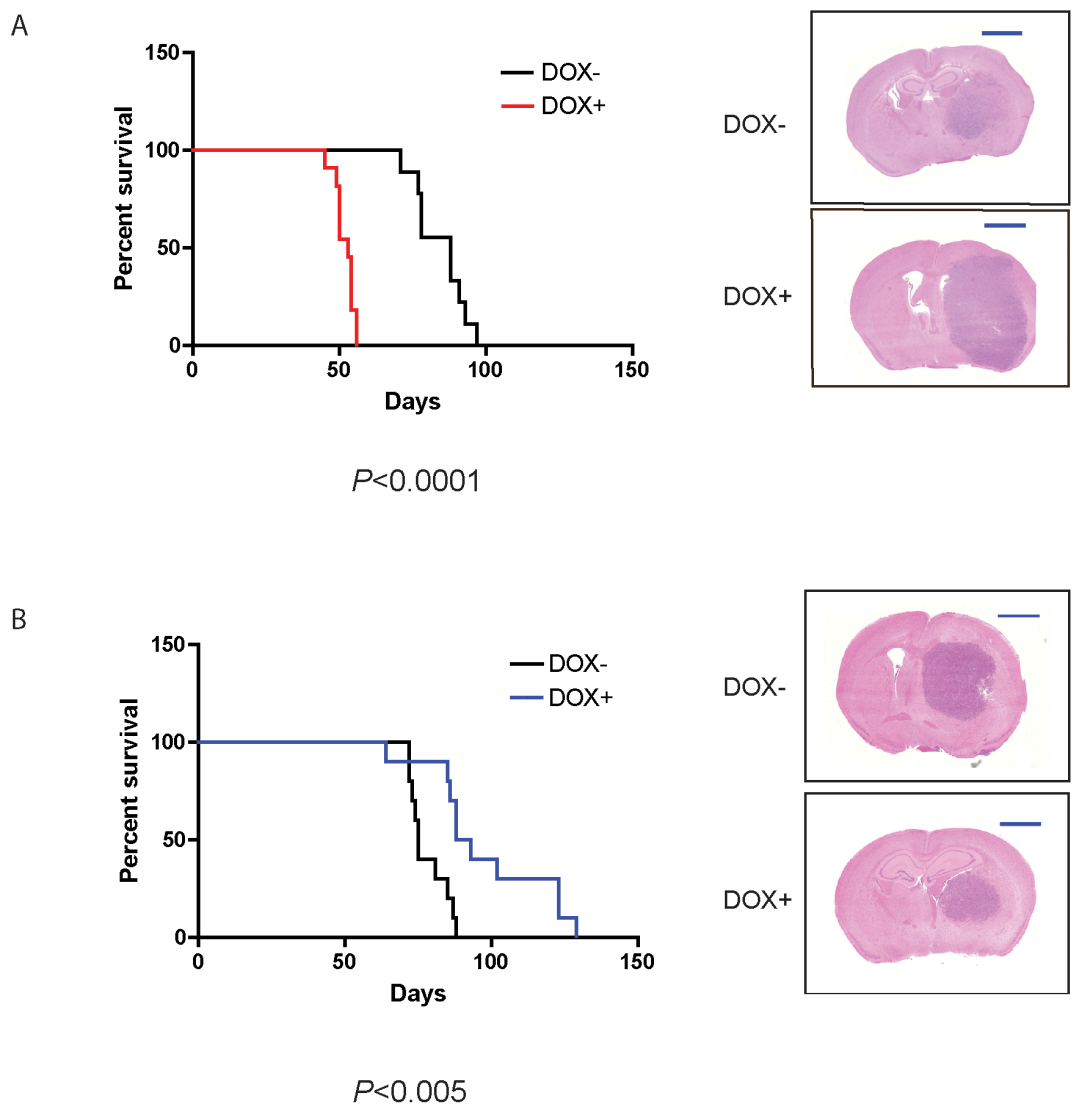
CA-Cdc42 expressing U251 MG cells decrease xenograft survival, whereas DN-Cdc42 expressing cells increase survival Kaplan-Meier survival curves of orthotopic mouse tumor xenografts of U251MG CA-Cdc42 **A.** and U251MG DN-Cdc42 **B.** treated + versus - doxycycline. CA-Cdc42 overexpression resulted in significantly decreased survival (*p* < 0.0001), whereas DN-Cdc42 overexpression resulted in significantly increased survival (*p* < 0.005). H&E staining of representative tumor xenografts. Scale Bar, 2mm.

Induction of CA-Cdc42 in U251MG cells dramatically reduced the survival of mice compared to controls; from 97 to 56 days for U251MG (*p* < 0.0001) (Figure 6A), and from 66 to 50 days for U87MG xenografts, although the latter did not reach statistical significance (*p* < 0.088) (Supplementary Figure S2A). Larger and more aggressive tumours were associated with earlier mouse mortality. On the other hand, induction of DN-Cdc42 in the xenografts prolonged the survival of mice, both in the U251MG and U87MG models (*p* < 0.005 and *p* < 0.05, respectively) (Figure 6B and Supplementary Figure S2B).

### Alteration of cell morphology in Cdc42-transfected glioma cells

**Figure 7:**
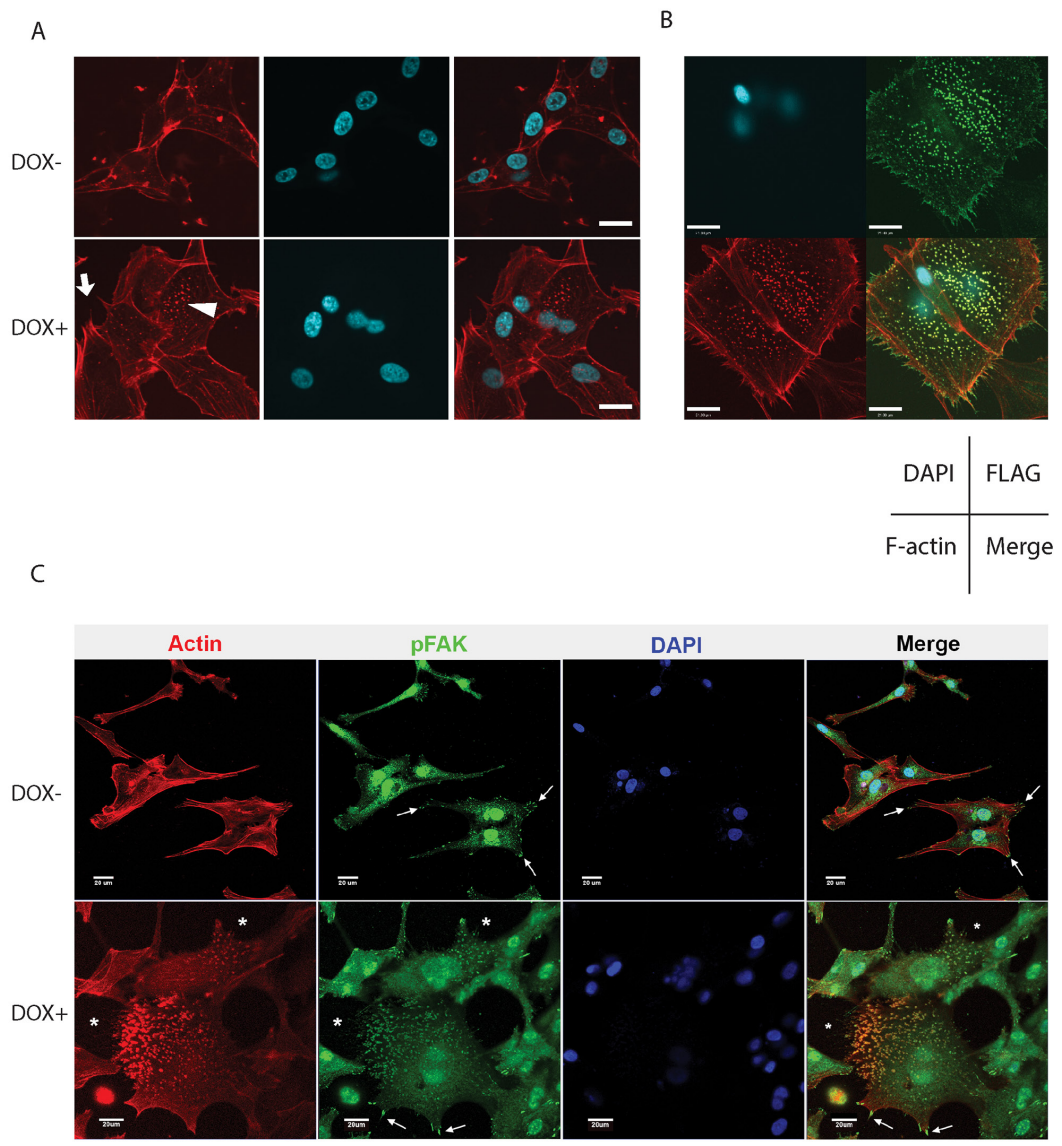
A. Change in morphology and actin cytoskeleton of U87MG CA-Cdc42 inducible cells F-actin and nuclei were stained with phalloidin 594 and DAPI, respectively. CA-Cdc42 expressing inducible cell clones show robust filopodia formation (arrow) and an increased number of focal adhesions (arrow head) compared to uninduced. Interestingly, the CA-Cdc42 cells have an increased cell surface area in the presence of doxycycline. Scale bar, 20μm. **B.** CA-Cdc42 localizes on the membrane, in filopodia, and within focal adhesion structures. A examples depicting immunofluorescence of U87MG cells stably expressing FLAG-CA-Cdc42 show Cdc42 (green) is co-localized at the cell membrane with filopodia (red, Phalloidin 594), and also accumulates at focal adhesions (green dots). The arrangement of images in each panel is indicated: nuclei (DAPI, blue); FLAG-Cdc42 (green); Phalloidin 594 (red). Scale bar, 21μm. **C.** pFAK co-localizes with focal adhesion structures. pFAK (green) co-localizes with focal adhesion structures (red) on CA-Cdc42 expressing U87MG. *; focal adhesion structures, arrow; pFAK localizes cell edges. Scale bar, 20 μm.

Immunofluorescence detection of F-actin filaments by phalloidin staining revealed the formation of numerous filopodia in U87MG cells upon activation of CA- and WT-Cdc42 expression (Figure 7A, Supplementary Figure S3). The overall surface area of the cells was increased compared to controls, together with the induction of prominent focal adhesion structures. These features were most pronounced in CA-Cdc42 cells and were not observed in DN-Cdc42 cells (Figure 7A, and Supplementary Figure S3). Induction of Cdc42-CA and -WT in U251MG similarly augmented filopodia formation and focal adhesion structures, although there was no cell surface area enlargement (Supplementary Figure S4). Control U251MG-DN cells demonstrated only minimal change in morphology with doxycycline (Supplementary Figure S4). To further determine if activated Cdc42 was involved in focal adhesion formation, we generated U87MG cells expressing FLAG-tagged CA-Cdc42 and studied their co-localization. Constitutively active Cdc42 localized within filopodia at the leading edge of cells (Figure 7B). In addition, CA-Cdc42 was widely distributed within focal adhesion structures (Figure 7B). We further investigated the composition of these focal adhesion structures by immunocytochemistry. Accordingly we used antibodies against phospho-FAK (pFAK), paxillin, and vinculin. Interestingly, only pFAK showed co-localization with the focal adhesion structures (Figure 7C). These results indicate that active Cdc42 has a strong effect on glioma cell morphology, by independently associating with two subcellular structures: filopodia and focal adhesion structures (pFAK).

### IQGAP1 co-localizes filopodia formation and focal adhesion formation and interacts with active form of Cdc42

**Figure 8:**
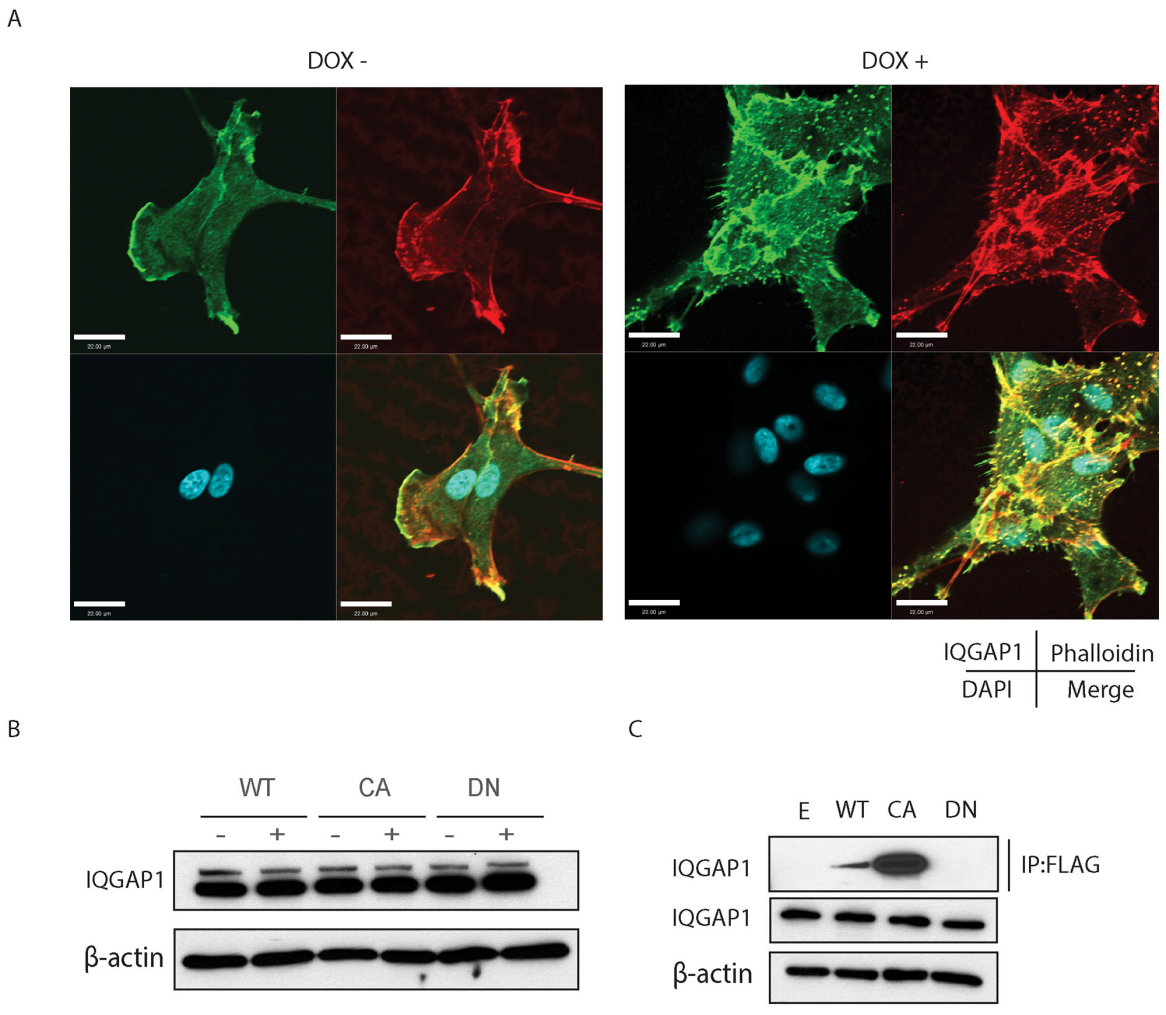
A. Confocal microscope images experiments demonstrate IQGAP1 co-localization to filopodia and focal adhesions Scale bar, 22μm. **B.** IQGAP1 expression in U87MG inducible cell lines. **C.** Pull-down assay with FLAG antibody in FLAG-Cdc42 expressing U87MG.

Mass spectrometry of Cdc42 immunoprecipitates of glioma cell lysates revealed a number of putative binding partners. Among these was IQ-domain GTPase-activating protein 1 (IQGAP1), a scaffold protein known to bind to Cdc42 and influence cell migration in normal and cancer cells (Supplementary Figure S5). IQGAP1 co-localized with filopodia formation and focal adhesion structures in the presence of doxycycline (Figure 8A right panel). Expression of activated Cdc42 does not change the expression of IQGAP1 (Figure 8B). Pull down assays were then performed using a FLAG antibody to show that IQGAP1 interacts only with the active form of Cdc42 (Figure 8C).

### Knockdown of IQGAP1 in CDC42-CA U251 glioma cells decreases migration, invasion, and proliferation

**Figure 9:**
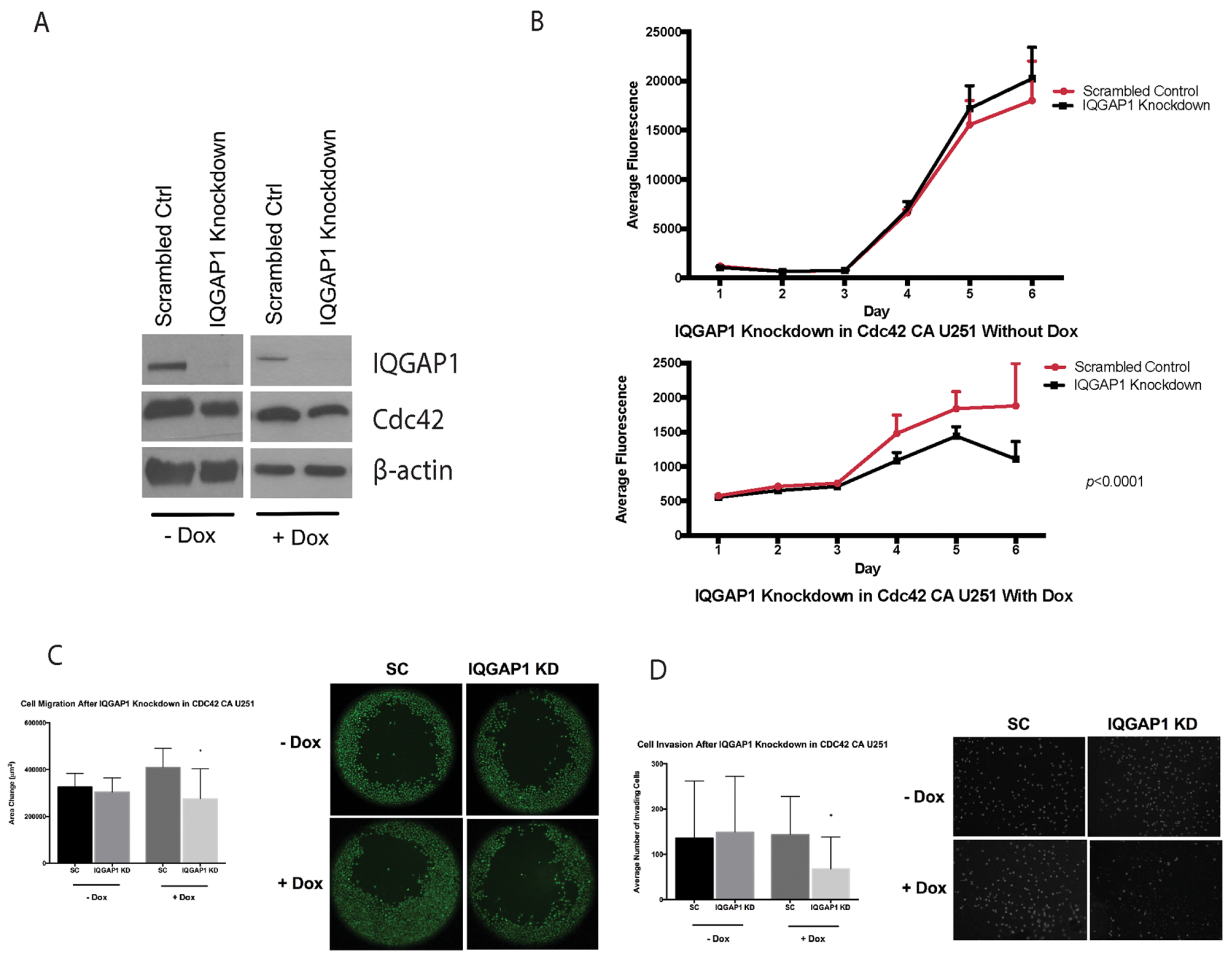
IQGAP1 siRNA on CA-Cdc42 inducible U251MG **A.** IQGAP1 knockdown with and without doxycycline. **B.** Proliferation assay after IQGAP1 knockdown. **C.** Migration assay after IQGAP1 knockdown *;*p*<0.05 **D.** Matrigel invasion assay after IQGAP1 knockdown. *;*p*<0.05

Knockdown of IQGAP1 in inducible U251MG glioma cells was performed to elucidate the functional role of IQGAP1. CA-Cdc42 U251MG cells were cultured with doxycycline for 3 days prior to IQGAP1 knockdown (Figure 9A). Interestingly, IQGAP1 specific knockdown significantly decreased cell migration (*p*<0.05), invasion (*p*<0.05), and proliferation (*p*<0.0001) compared to scrambled control in the inducible U251MG cells with doxycycline (Figure 9B–9D). However, no significant differences in migration (*p*=0.24), invasion (*p*=0.57), and proliferation (*p*=0.35) were detected without doxycycline in IQGAP1 knockdown group in comparison to scrambled control (Figure 9B–9D).

### Cdc42 expression correlates with outcome in patients with GBM

**Figure 10:**
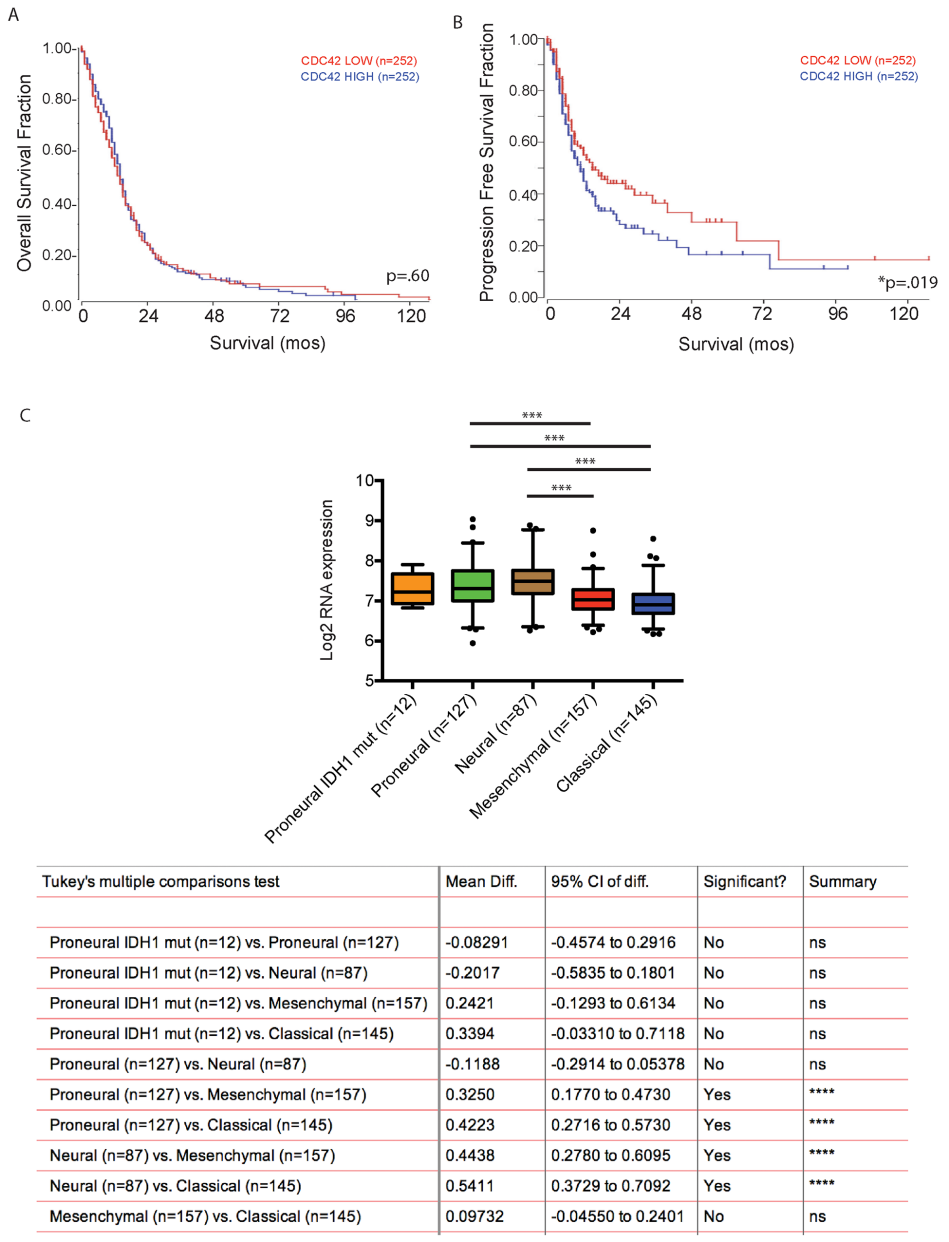
Cdc42 expression level does not predict patient survival with GBM but is associated with poor progression free survival **A.** Kaplan-Meir Survival curve analysis comparing overall survival of Cdc42 high versus low expressing patients in TCGA dataset. High Cdc42 expression is not associated with overall survival of patients with GBM. (*p* = 0.60) **B.** In contrast, high Cdc42 expression is significantly associated with poor progression free survival (*p* < 0.05). **C.** Cdc42 expression is significantly higher in pro-neural and neural subtypes of GBM compared to classical and mesenchymal subtypes.

We then queried The Cancer Genome Atlas GBM patient data set, and used a median mRNA expression cutoff to correlate Cdc42 expression levels with patient clinical outcome. High Cdc42 expression was associated with poorer progression free survival (*p* < 0.019), but not overall survival (Figure 10A and 10B). As GBM is comprised of several molecular subgroups (Proneural, Neural, Classical, Mesenchymal, and the G-CIMP positive subgroup), each associated with unique transcriptomic, genetic, epigenetic and clinical features, we next explored subgroup specific Cdc42 expression patterns in GBM samples. Cdc42 RNA expression was significantly higher in the proneural and neural subgroups compared to the classical and mesenchymal subgroups of GBM in the TCGA dataset (Figure 10C).

## DISCUSSION

In this study, we have examined the role of Cdc42 on the morphology, growth pattern and tumorigenicity of human GBM cells. We show that activation of Cdc42 expression enhances the migration and invasion of human glioma cells in an orthotopic glioma model. We also demonstrate the colocalization of active Cdc42 with IQGAP1 and phosphorylated FAK.

Cell migration requires the formation and extensions of membrane protrusions, including filopodia. The driving force for the formation and extension of such protrusions is the polymerization of actin [17]. F-actin staining revealed that glioma cells expressing CA-Cdc42 demonstrate a greater number of filopodia than glioma cells expression WT- and DN-Cdc42. CA-Cdc42 expressing glioma cells also demonstrated an increased number of phosphorylated focal adhesion complexes. Focal adhesions form in part through a dynamic interplay between Rho-GTPase expression and cell contact with the ECM [18]. ECM binding to cell surface receptors promotes the activity of the Rho-GTPases which control the contraction of F-actin fibers through proteins such as Arp2/3 and myosin [17]. During the process of cell spreading, the cell first attaches to the ECM followed by the extension of cell protrusions. If adhesion to the ECM is impaired, cells are incapable of initiating cell protrusion [17, 19]. Hence, the formation of focal adhesion structures is a critical step in the initiation of cell migration and invasion. Enhanced formation of focal adhesions induced by active Cdc42, as demonstrated in our study, may stimulate cell migration and invasion.

FAK is a non-receptor cytoplasmic-tyrosine kinase that plays a critical role in the migration and proliferation of several cell types through the activation of different signaling pathways [20–22]. There have been several prior reports showing high FAK expression in glioblastoma [23–26]. FAK is also known to enhance cell migration [22]. Our results demonstrated that CA-Cdc42 increase focal adhesion structure, which are co-localized to pFAK. This co-localization suggests that active Cdc42 may regulate pFAK distribution enhancing glioma migration and invasiveness.

Interestingly, in our study the expression of Cdc42 had little impact on cell proliferation, regardless of Cdc42 activation status. This result differs somewhat from previous reports which have shown that Cdc42 is a regulator for cell cycle progression in fibroblasts [27]. In addition, silencing of Cdc42 has been shown to inhibit cell proliferation in neuroblastoma cells [28]. However, Gao et al. demonstrated that proliferation and migration phenotypes can switch under certain conditions [29]. In theory, the proliferative and invasive phenotypes may be temporally and mutually exclusive. For example, highly invasive glioma cells have a lower proliferation rate during locomotion, whereas highly proliferative cells are less invasive [30]. Our results showed that induction of WT- and CA-Cdc42 increased migration and invasion of glioma cells, but that their proliferation was decreased.

We have demonstrated co-localization of IQGAP1 and the activated form of Cdc42 in focal adhesions. IQGAP1 is scaffold protein that has been shown to assemble the actin cytoskeleton through binding to Cdc42, a process which can control invasion and migration by negatively regulating E-cadherin-based cell-cell interaction [31, 32]. Interestingly, it has been shown that a reduction in IQGAP1 expression is closely linked to long-term survival of patients with GBM [33]. In another study, IQGAP1 was shown to be a marker of nestin positive amplifying neural progenitor cells [34] Hu et al. demonstrated that IQGAP1 is required for activation of Rac1 in a signaling pathway shown to be crucial for invasion of glioma cells [35]. In other cancer cell systems, it has been shown that the active form of Cdc42 is capable of binding to IQGAP1 to increase cancer migration and carcinogenesis [36, 37]. In our study, CA-Cdc42 was shown to bind IQGAP1, but forced expression of CA-Cdc42 did not alter the expression level of IQGAP1. Our results demonstrated that only CA-Cdc42 recruits and binds to IQGAP1, and this interaction can promote actin cytoskeletal rearrangement that may lead to increased invasion and migration of glioma cells.

In order to investigate the functional role of IQGAP1 on CA-Cdc42 expressing cells, we performed IQGAP1 knockdown studies. These studies showed that IQGAP1 specific knockdown decreases proliferation, migration, and invasion only in the context of increased expression of CA-Cdc42. These results suggest that CA-Cdc42 expression is closely correlated with IQGAP1 and enhances the migration and invasiveness of glioma.

We also interrogated the clinical significance of Cdc42 expression in reference to the TCGA dataset. High Cdc42 expression was associated with a worse progression free survival, suggesting that high Cdc42 expression may be linked to GBM recurrence. From a prognostic perspective, however, high Cdc42 expression was not associated with worsened overall survival. One possible explanation for this lack of correlation is that the activation status of Cdc42 has not been taken into account with such database queries. It is also important to note that the gene expression analyses housed on the TCGA are derived from whole brain tumor specimens, and may not be representative of the remodeling activity present at the invasive, leading edge of the tumor. Recent studies have shown that localized alterations in gene and protein expression in actively invading glioma cells may be more responsible for their migratory properties than glioma cells within the tumor core [38–41]. Jarzynka et al. showed this for the bipartite Rac-GEF, ELMO1 and Dock180, which were upregulated in the actively invading glioma cells at the invasive areas of the tumor rim, but not in the central regions of the tumor [40].

N-WASP and p21-activated kinase (PAK) are downstream effectors of Cdc42 [42, 43]. The active form of Cdc42 binds to N-WASP or PAK and promotes actin cytoskeletal polymerization or promoting PAK autophosphorylation, respectively [42, 43]. These molecular targets which bind to CA-Cdc42 may facilitate the indirect localization of activated Cdc42 in human glioma samples. Such studies may prove useful in the future when investigating the relationship between activated Cdc42 expression in human samples and clinical datapoints such as progression-free or overall survival.

Intriguingly, our results showed that DN-Cdc42 significantly decreases migration and invasion in U87MG *in vitro* as well as significantly increases mouse xenograft survival of U251MG *in vivo*. These results suggest that suppression of Cdc42 activity can lead to a decrease in glioma invasion and migration. Accordingly in our future studies, we plan to examine the effects of the various commercially available Cdc2 pharmacological inhibitors [44–47]. There are a number of Cdc42 GEFs that have been previously described [48]. It is conceivable that these GEFs are involved in the regulation of oncogenic Cdc42 activity in glioma cells. As a result, it is possible to consider that glioma invasion could also be significantly reduced through the inhibition of these Cdc42 GEFs [5, 41, 49].

In conclusion, our study provides new insights into the functional significance of Cdc42 activity and human GBM migration and invasiveness. In addition, we have demonstrated a role for the binding of and interaction between Cdc42 and IQGAP1 and co-localization of pFAK in these cellular processes. It is our hope that the use of targeted Cdc42 inhibitors may be of value as adjunctive therapies in human GBM to decrease its aggressive behavior [18].

## MATERIALS AND METHODS

### Cell lines and culture conditions

The human malignant glioma cell lines, A172, U118MG, U251MG, U87MG, and LN229 were obtained from American Type Culture Collection (Manassas, VA). Cell lines were maintained in Dulbecco's modified Eagle's medium (DMEM) supplemented with 10% heat-inactivated fetal bovine serum (FBS) in a humidified atmosphere containing 5% CO2 at 37°C. The cells were routinely inspected for mycoplasma.

### Western blot analysis

Total cell lysates were prepared by harvesting cells in RIPA lysis buffer (Sigma-Aldrich, St, Louis, MO) with a protease inhibitor cocktail (Roche Diagnostics, Indianapolis, IN). Protein concentration was determined using the Pierce BCA Protein Assay Kit (Thermo Scientific, Rockford, IL). Protein extracts were mixed with 6X SDS sample buffer (Tris pH 6.8, 1.7% SDS, glycerol and β-mercaptoethanol), and the cell lysates were resolved on 12% SDS-polyacrylamide gels of 1.5 mm thickness. Proteins were then transferred onto polyvinylidene Fluoride Transfer Membranes (Pall Corporation, Pensacola, FL), and subsequently blocked with 5% skim milk in TBST (20mM Tris aminomethane, 150mM NaCl and 0.05% Tween 20; pH 7.4) for 1 hr at room temperature. The membranes were incubated overnight at 4 °C with primary antibodies, then at room temperature for 1 hr with the secondary antibodies, either horseradish peroxidase-conjugated goat anti-rabbit or anti-mouse immunoglobulin G antibody (1:5000; Cell Signaling Technology), and bound primary antibodies were visualized using Western Lightning Plus-ECL (PerkinElmer Inc., Waltham, MA). The primary antibodies used in this study were as follows: anti-Cdc42 (Rabbit polyclonal; 1:000; Cell Signaling) anti-Cdc42 (Mouse monoclonal; 1:200; Santa Cruz), anati-IQGAP1 (Rabbit polyclonal; 1:2000; Abcam) and anti-β-actin (Rabbit monoclonal; 1:20,000; Cell Signaling).

### Site-directed mutagenesis

Constitutively active-Cdc42G12V (CA), and dominant negative-Cdc42T17N (DN) were generated by Site-directed mutagenesis QuikChange II XL kit (Agilent Technologies, Santa Clara, CA) with primers 5′-GTGTTGTTGTGGGCGATGTTGCTGTTGGTAAAACATG-3′ (underlined T is G in the wild-type sequence) and 5′-GTGGGCGATGGTGCTGTTGGTAAAAATTGTCTCCTGATAT-3′ (underlined AT is CA in the wild-type sequence), respectively. The full-length sequence of mutated Cdc42 was verified by DNA sequencing by ACTG Corporation (Toronto, Canada).

### siRNA treatment

A small interfering RNA (siRNA) quadriplex against human Cdc42 or IQGAP1 ON-TARGET plus SMARTpool was purchased from Dharmacon (Dharmacon, Thermo Scientific, Waltham, MA). For monitoring off-target effects of RNA interference, ON-TARGET plus non-targeting pool (Dharmacon) was used. Transient transfection of siRNA was performed using HiPerfect (QIAGEN, Toronto, Canada), according to the manufacturer's protocol. Briefly, 5×10^5^ cells were seeded on a 6 cm^2^ dish in DMEM containing 10% of FBS one day prior to transfection. On the following day, 256 ng of siRNA and 20 ml of HiPerfect were diluted in 100 ml of Opti-MEM (GIBCO) medium and incubated for 10 min at room temperature. After incubation, the siRNA transfection complexes were added to the cells on a 6 cm^2^ dish containing 4 ml of DMEM with 10% FBS.

### Preparation of U87MG and U251MG clones conditionally expressing Cdc42

The Tet-On^®^ 3G Inducible Expression System containing pLVX-TRE3G-IRES and pLVX-Tet3G vectors was purchased from Clontech (Mountain view, CA). The Cdc42 construct (pCMVXL5-Cdc42) was generated by SIDNET at The Hospital for Sick Children. Briefly, the *Cdc42* open reading frame was cloned into the pLVX-Tet3G vector via *BamHI* and *NotI* sites and packaged into lentiviruses [50]. Cells infected with viruses were selected in medium containing puromycin at 0.4 μg/ml for U87MG and U251MG. Doxycycline (Sigma-Aldrich) at 500ng/ml was used to induce Cdc42 expression in cell culture. Cdc42 activation assay and western blot analysis were performed to verify the construct expression level.

### Preparation of U87MG clones expressing FLAG-Cdc42

Inserts from plasmids containing WT-, CA-, DN-Cdc42 open reading frames were subcloned into the pFLAG-CMV-10 vector (Sigma-Aldrich). To generate stable transfectants, the constructs were transfected into the U87MG cell lines using X-tremeGENE9 (Roche Diagnostics) as per the manufacturer's protocol. The transfected cells were then seeded on 10 cm^2^ plates and kept under G418 sulfate selection medium containing 600mg/ml of Geneticin (GIBCO, Green Island, NY). FLAG-Cdc42 expression was verified by the activation assay and western blot analysis.

### Preparation of LN229 human glioma clones conditionally expressing Cdc42

Plasmids carrying eGFP fused to the N-terminus of CA-Cdc42 (Q61L) and DN-Cdc42 (T17N) mutants were obtained from Addgene. Inserts were subcloned into the pTRE-2 plasmid (Clontech), the resulting constructs and pcDNA3 (at a 10:1 ratio) were used to co-transfect LN229-L16 tet-on cells (a clone of LN229 glioma cells stably expressing rtTA [51]) with Effectene (QIAGEN). Stably transfected clones were selected in the presence of 600 μg/ml G418 and characterized for doxycycline (1 μg/ml)-inducible expression of eGFP by immunofluorescence and immunoblotting.

### Cdc42 activation assay

To quantify the amount of activated Cdc42 in cells, a Cdc42 pulldown assay was performed (EMD Millipore, Billerica, MA). Approximately 8×10^5^ of cells were seeded in 10 cm^2^ plates and incubated over night, and then cultured for 3 days in the presence of doxycycline. Subsequently, cells were lysed in RIPA buffer containing protease inhibitor cocktail and protein concentrations were determined. To 500 μg of each sample in a volume of 500 μl, 10 μl of PAK1-PBD agarose beads (EMD Millipore) were added and incubated for 45 min at 4°C with gentle rotation. Beads were washed three times with 500 μl of the cold lysis buffer, resuspended in 20 μl of 2X SDS-sample buffer, boiled and loaded onto a 12% SDS-PAGE gel. The amount of activated and total Cdc42 in each sample was analyzed with the rabbit anti-Cdc42 antibody described above.

### Cell viability assay with MTS assay

The cell viability of A172, U87, and U118 after Cdc42 specific siRNA was determined using the MTS (3-(4,5-dimethylthiazol-2-yl)-5-(3-carboxymethoxyphenyl)-2-(4-sulfophenyl)-2H-tetrazolium) assay (Promega, Madison, WI). Glioma cells were either untransfected, or transfected with control or Cdc42 siRNA cultured for 72 hrs and then incubated for 1 hr with MTS. Absorbance was detected at 490 nm.

### Cell proliferation assay with alamar blue

U87 MG and U251 MG Tet-on inducible cell lines were grown in tissue culture plates in DMEM with 10% FBS with or without doxycycline. Three days after treatment, the cells were seeded on 96-well plates at a density of 1000 cells in 100 μl of culture medium containing 10% of FBS with or without doxycycline per well. After 2 hr incubation with 10 μl of Alamar Blue Cell Viability Assay Reagent (Thermo Scientific, Rockford, IN) per well, the number of viable cells was measured using the VERSA max microplate reader (Molecular Devices, Sunnyvale, CA) at 550 and 570 nm wavelength daily for six days after seeding cells.

### Radial cell migration assay

Cell migration was assessed by the microliter-scale radial monolayer assay with some modifications to the previously described method [52, 53]. Briefly, 10-well Teflon-coated glass slides (CSM Inc., Phoenix, AZ) were coated at 4°C overnight with 0.1% BSA in PBS. Cdc42 transient siRNA transfected cells which were collected 48 hrs after siRNA transfection were seeded through a cell sedimentation manifold (CSM Inc.) at 3000 cells per well to establish a circular confluent monolayer at the center of the ECM coated well. After 24 hrs of incubation, manifolds were removed, and a circle best-fit circumscribing the cells was drawn as baseline data. The cells were allowed to migrate for 24 hrs in culture medium, and another circle circumscribing the newly migrated cells was drawn. The average migration rate (mm/24 hrs) was determined by the increase of the diameter of the circle beyond the baseline diameter of the cells during a 24 hours period. Images were taken with a stereomicroscope (Leica fluorescence stereomicroscope; Leica Microsystems, Bannockburn, IL) equipped with a CCD camera and analyzed using Volocity image analysis software (PerkinElmer Inc.). Experiments were repeated three times with five replicates.

### The Oris^TM^ cell migration assay

The Oris^TM^ Cell Migration Assay plate (Platypus technologies, Madison, WI) was used to test the migration of glioma cells. Before the assay, cells were pretreated with 500 ng/ml doxycycline for 3 days to induce Cdc42 expression. Approximately 4×10^4^ of U87MG and 3×10^4^ cells of U251MG cells were seeded in each well. After overnight incubation, stoppers were removed. The plate was incubated for 24 hrs at 37°C in a 5% carbon dioxide humidified chamber to allow the cells to migrate towards the center of the well. After 24 hrs of incubation, the cells were labeled with the Calcein AM cell-permeant dye (Life Technologies) and the area over which the cells migrated was quantified using Volocity 6.3 software (Perkin Elmer). Experiments were repeated three times in six replicates.

### Two-dimensional cell invasion assay

Glioma cell invasion was assessed using BD BioCoat Matrigel Invasion Chambers consisting of Transwell-precoated Matrigel membrane filter inserts with 8 μm pores in 24-well tissue culture plates (BD Biosciences, Bedford, MA). Approximately 4×10^4^ of cells pretreated with or without 500 ng/ml of DOX were collected 3 days after treatment and seeded onto the top of the chamber in DMEM with 0.1% FBS, and the bottom chamber was filled with DMEM with 10% FBS as a chemoattractant. The cells were incubated for 12 hrs at 37°C in a 5% carbon dioxide humidified incubator to allow them to invade through the filter membrane. After 12 hrs of incubation, non-invading cells were removed from the upper surface of the membrane using cotton swabs, and the filter membranes were fixed with 4% paraformaldehyde for 15 min at room temperature. Subsequently, the filter membranes were mounted on glass slides using Vectashield with DAPI (Vector Laboratories Inc., Burlingame, CA) for nuclear staining. Fluorescence was visualized and images were taken using a fluorescence microscope. The number of cells that had invaded through the membrane in six different microscopic fields per filter was quantified using Volocity software. Experiments were repeated three times in triplicate.

### Three-dimensional spheroid invasion assay

LN229 cells were cultured in 25cm^2^ flasks with 5 ml DMEM medium (Cellgro 10-013-CV) supplemented with 10% FBS, pen/strep and L-glutamine. Approximately 3 - 4,000 cells per well were transferred into 96 well plates pre-coated with solidified agarose (1%) to generate spheroids at 37°C in a tissue culture incubator. After 7-10 days, once spheroids reached a size of 150-200 uM in diameter, doxycycline (1μg/ml) was used to induce GFP-Cdc42 expression for 24 hrs. Spheroids were then transferred to a 35 mm glass bottom imaging dish (In Vitro Scientific. Cat# D35-14-1.5-N) and embedded within BD Matrigel Basement Membrane Matrix (BD Biosciences, Cat# 356237). Spheroids within the imaging dish were bathed in complete DMEM medium and imaged using a Perkin Elmer Spinning Disc Confocal Microscope (UltraViewRS, Perkin Elmer) with a 10x Plan-Neofluar objective (NA=0.3) every 5 min at 37 °C with 5% CO_2_ as described (http://www.ncbi.nlm.nih.gov/pubmed/22688514). Z-stacks were acquired for each time point and data was compiled and analyzed using Volocity software.

### Immunofluorescence and confocal microscopy

Doxycycline-inducible cells were seeded at a density of 4×104 in glass bottom plates (Greiner Bio-One International AG, Frickenhausen, Germany) and cultured with 500 ng/ml of doxycycline for 3 days. Cells were washed with 1X PBS three times and fixed with 4% PFA for 15 min at room temperature. Cells were then permeabilized for 10 min with 0.25% Triton X-100 in PBS at room temperature. Non-specific binding was blocked by 0.3% goat serum in PBS for 1 hr at room temperature. Subsequently, for FLAG-Cdc42 expressing cells and Cdc42 expressing inducible cells, cells were incubated with anti-FLAG antibody (1:1000; SIGMA), anti-IQGAP1 antibody (1ug/ml; Abcam), and for U87MG CA-inducible cells, anti-pFAK antibody (5ug/ml; Abcam) at 4°C overnight, respectively. After washing with PBS three times, cells were incubated with Alexa Fluor 488-conjugated secondary antibody (1:500; Molecular Probes) or Phalloidin 594 (1:500, Molecular Probes) together with 4′, 6-diamidino-2-phenylindole dihydrochloride (DAPI) for 1h at room temperature. Fluorescence signal was visualized and photographs were taken using a Zeiss Axiovert 200M Spinning Disc confocal microscope (Carl Zeiss Canada, Toronto, ON, Canada) equipped with a Hamamatsu Back-Thinned EM-CCD camera (Hamamatsu Corporation, Bridgewater, NJ).

### *In vivo* orthotopic mouse glioma xenograft studies

All mouse studies were approved and performed in accordance with the policies and regulations of the Institutional Animal Care Committee of the Hospital for Sick Children, in Toronto. Athymic NOD scid gamma mice (The Jackson Laboratory, Bar Harbor, ME) underwent intracranial (right frontal lobe) implantation of 5×10^4^ tumor cells in 2.5 μl of PBS using a Hamilton syringe on a stereotaxic frame. Before the intracranial injection, cells were treated with or without doxycycline for 3 days. Doxycycline was administered in the animal's food (Harlan Laboratories, Madison, WI) immediately after surgical implantation of tumor cells. Kaplan-Meier survival curves were established and statistical analyses performed with GraphPad Prism5.

### FLAG pull-down assay

FLAG-Cdc42 expressing U87MG cells were seeded on the 10 cm^2^ plate and incubated until cells grew to 80% confluence. Cells were lysed in immunoprecipitation buffer (150mM NaCl, 50mM HEPES, 10% glycerol, 1% Triton X-100, 2mM EDTA, protease inhibitor and phosphatase inhibitors) at 4°C. Immunoprecipitation was performed using anti-FLAG conjugated magnetic beads (SIGMA) at 4°C over night. After incubation, the beads were washed with ice-cold PBS three times. Bound proteins were separated by SDS-PAGE followed by immunoblotting using specific antibodies.

**Mass spectrometry of Cdc42 binding partners** (See Supplementary Methods)

### Analysis of Cdc42 expression levels in relation to human GBM subtype and patient survival

GBM gene expression data for analysis of subtype differences was obtained from the TCGA data portal (https://tcga-data.nci.nih.gov/tcga/) accessed on July 10 2015. 528 GBM samples with subtype information as defined by the Verhaak et al. study were selected for Cdc42 subgroup analysis [54]. Briefly, Affymetrix gene expression profiles on the U133A platform level 3 normalized data were downloaded. We compared normalized Cdc42 gene-expression values amongst subgroups. Analysis of variance (ANOVA) was conducted for multi-group comparisons followed by a post-Tukey test (to identify differences among sub-groups). Significance was defined as *p* < 0.05. Survival analysis on Cdc2 expression levels were performed in the Cancer Genome Atlas (TCGA) GBM data set, comprising 504 patients with survival and progression free survival information. A median RNA expression cutoff was selected for both survival and progression free survival analysis (above the median was identified as high expression and below the median was selected as low expression). Patients were censored if RNA expression, survival or progression free survival information was unavailable. Significance was set at *p* <0.05.

## SUPPLEMENTARY MATERIALS FIGURES AND VIDEOS








