# Supplementary material for: A role for activated Cdc42 in glioblastoma multiforme invasion
Source: Oncotarget. 2016 Jul 29;7(35):56958–75. doi: 10.18632/oncotarget.10925 (PMC5302965; doi:10.18632/oncotarget.10925)
Supplement: Supplementary file 1 [file oncotarget-07-56958-s001.pdf]

A role for activated Cdc42 in glioblastoma multiforme invasion

SUPPLEMENTARY MATERIALS AND METHODS

In vivo orthotopic mouse U87MG glioma xenograft studies

All mouse studies were approved and performed in accordance with the policies and regulations of the Institutional Animal Care and Use Committee of the University of Toronto and the Hospital for Sick Children, in Toronto. Athymic NOD scid gamma mice (The Jackson Laboratory, Bar Harbor, ME) underwent intracranial (right frontal lobe) implantation of 2.5×10^5 tumour cells in 2.5 µl of PBS using a Hamilton syringe on a stereotaxic frame. Doxycycline was administered in the animal's food (HarlanLaboratories, Madison, WI) 7 days after surgical implantation of tumour cells. Kaplan- Meier survival curves were established and statistical analyses performed with GraphPad Prism5.

Mass spectroscopy – samples run on the Q-Exactive-

Samples were analyzed on a Orbitrap analyzer (Q-Exactive, ThermoFisher, San Jose, CA) outfitted with a nanospray source and EASY-nLC nano-LC system (ThermoFisher, San Jose, CA). Lyophilized peptide mixtures were dissolved in 0.1% formic acid and loaded onto a 75µm x 50cm PepMax RSLC EASY-Spray column filled with 2µM C18 beads (ThermoFisher San, Jose CA) at a pressure of 800 Bar. Peptides were eluted over 60 min at a rate of 250nl/min using a 0 to 30% acetonitrile gradient in 0.1% formic acid. Peptides were introduced by nano-electrospray into the Q-Exactive mass spectrometer (Thermo-Fisher). The instrument method consisted of one MS full scan (400–1500 m/z) in the Orbitrap mass analyzer with an automatic gain control (AGC) target of 1e6, maximum ion injection time of 120 ms and a resolution of 70,000 followed by 10 data-dependent MS/MS scans with a resolution of 17,500, an AGC target of 1e6, maximum ion time of 120ms, and one microscan. The intensity threshold to trigger a MS/MS scan was set to 3.3e4. Fragmentation occurred in the HCD trap with normalized collision energy set to 27. The dynamic exclusion was applied using a setting of 10 seconds.

3D Spheroid proliferation assay

Proliferation in 3D culture was measured using Cultrex 3D Spheroid Colorimetric Proliferation/Viability Assay (Trevigen). Briefly, cells (5,000/well) were plated in triplicates in a round-bottom 3D Culture Qualified 96 well Spheroid Formation Plate in Spheroid Formation Extracellular Matrix (ECM), allowed to form spheroids for 72 hrs, and treated with appropriate concentrations of doxycycline for indicated times using the MTT assay as recommended by the manufacturer. The MTT assay was performed on spheroids for 72 hr without any treatment. Absorbance at 570 nm was corrected for the background which was comprised of ECM alone.

SUPPLEMENTARY FIGURES AND VIDEO

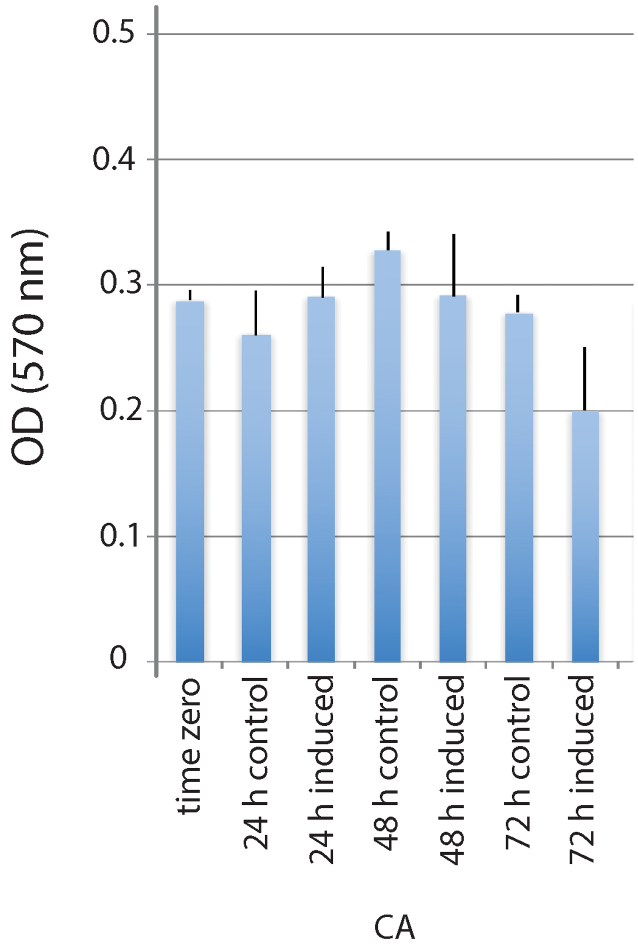

Supplementary Figure S1: (A) 3D cell culture proliferation for CA-Cdc42 LN229 cells as measured using the MTT assay.

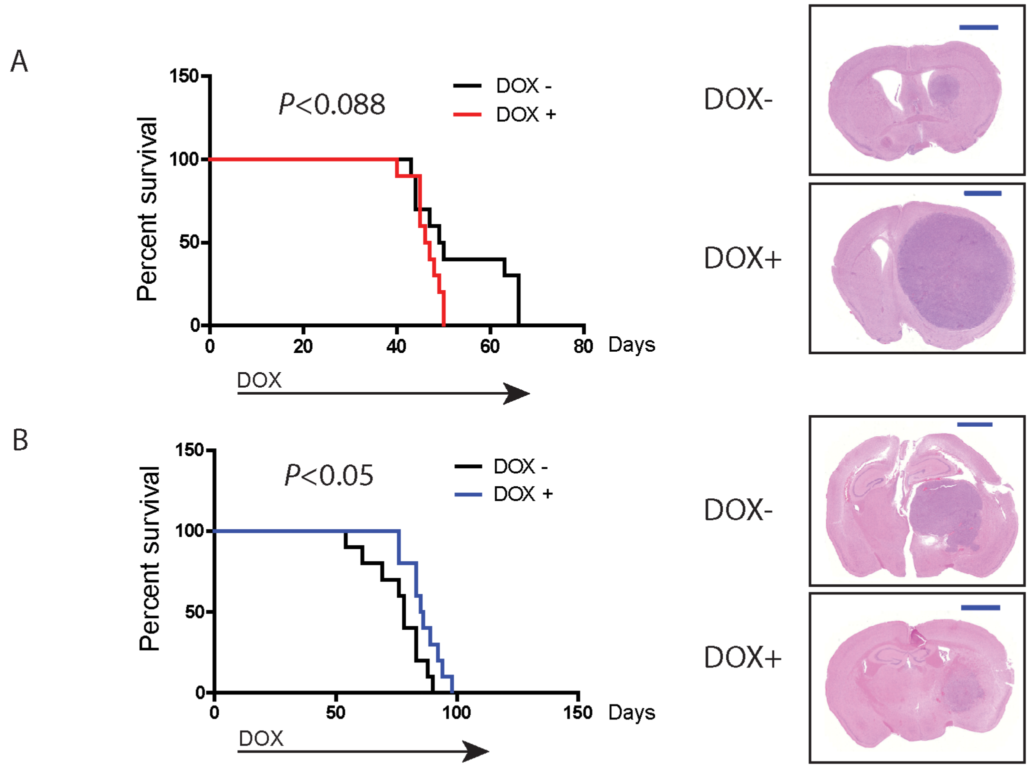

Supplementary Figure S2: DN-Cdc42 expressing U87MG cells increase survival in the xenograft model. A. Kaplan-Meier survival curves of orthotopic mouse tumourxenografts of U87MG CA-Cdc42 treated with doxycycline versus CA-Cdc42 withoutdoxycycline. Although doxycycline induced overexpression of CA-Cdc42 resulted in atrend towards the decreased survival of mice, there was no statistical significance. H&Estaining of the tumour xenografts revealed larger tumours in CA-Cdc42 expressing cells. **B.** Kaplan-Meier survival curves of orthotopic mouse tumour xenografts of U87MG DN-Cdc42 treated with doxycycline versus DN-Cdc42 without doxycycline.Doxycycline induced overexpression of DN-Cdc42 resulted in significantly increasedsurvival of mice ($p < 0.05$). Scale Bar, 2mm.

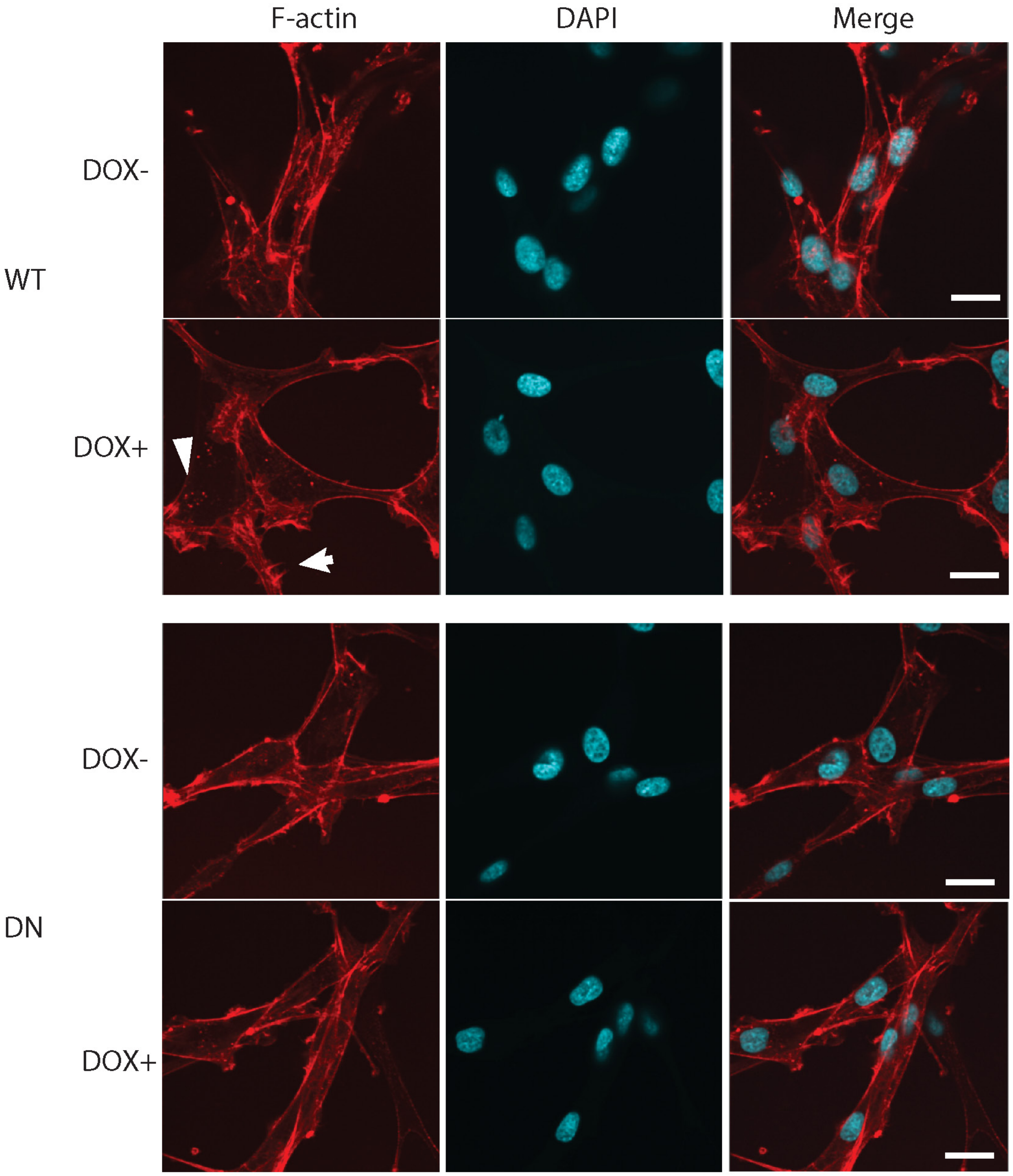

Supplementary Figure S3: Change in morphology and actin cytoskeleton of U87MG inducible cell clones. U87MG inducible cell clones were seeded onto plates and grownwith standard growth medium with (+) or without (-) doxycycline for 72 hours. F-actinand nuclei were stained with phalloidin 594 and DAPI, respectively. WT-Cdc42expressing inducible cell clones demonstrate filopodia formation (arrow) and focaladhesion structures (arrow head) in the presence of doxycycline. DN-Cdc42 expressinginducible cell clones do not demonstrate filopodia formation or focal adhesion structures both with and without doxycycline. Scale bar, 20µm.

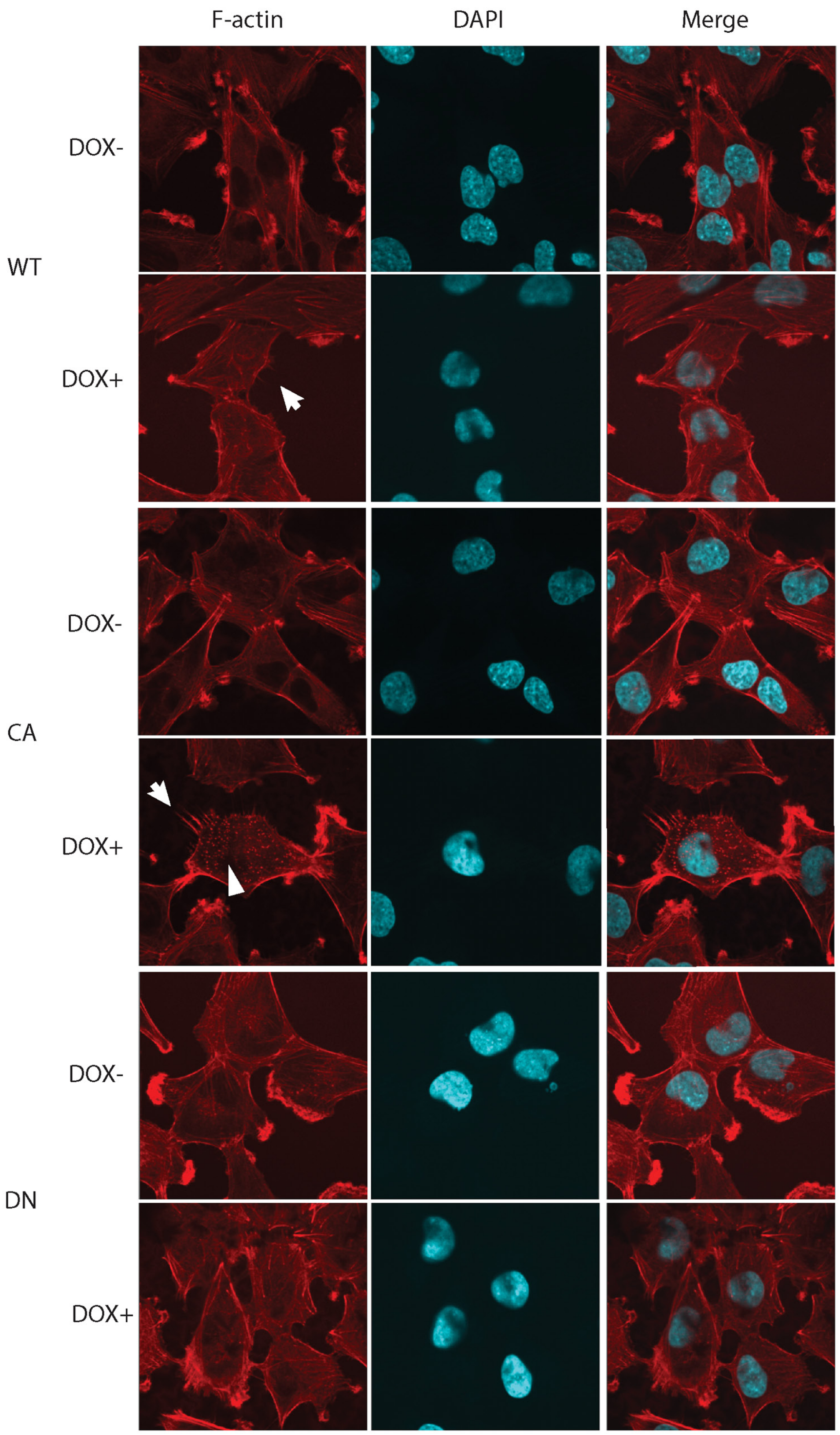

Supplementary Figure S4: Change in morphology and actin cytoskeleton of U251MG cell clones expressing Cdc42. U251MG cells with doxycycline inducible Cdc42 wereseeded onto plates and grown in standard growth medium with (+) or without (-) doxycycline for 72 hr. F-actin and nuclei were stained with phalloidin 594 and DAPI,respectively. WT-Cdc42 expressing cells demonstrated filopodia formation (arrow) andfocal adhesion structures (arrowhead) in the presence of doxycycline. CA-Cdc42expressing cells showed robust filopodia formation and an increased number of focaladhesion structures compared to the WT-Cdc42 induced cells. These features were absentin DN-Cdc42 expressing cells with and without doxycycline.

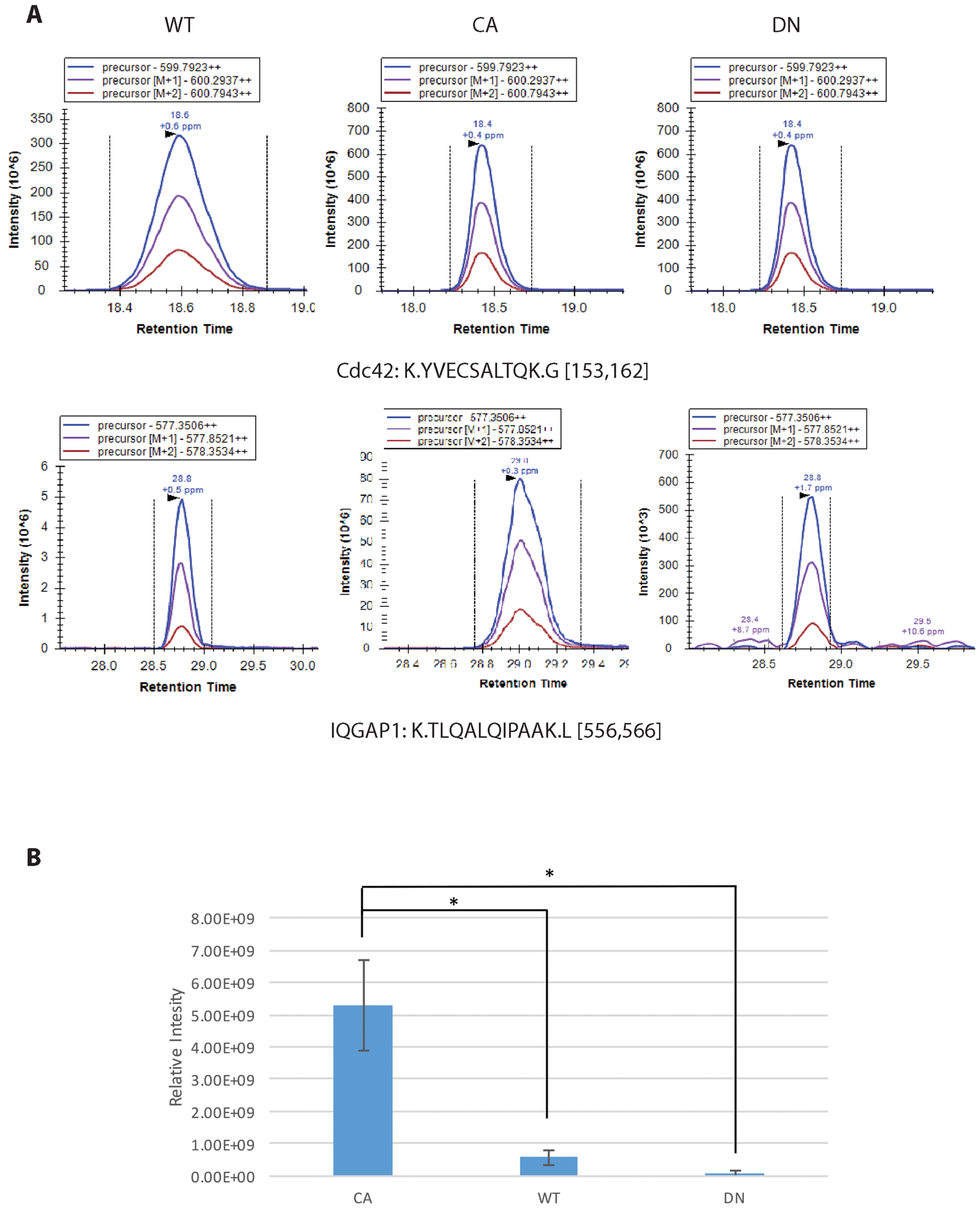

Supplementary Figure S5: U87 FLAG-Cdc42 Mass Spectroscopy A. Representative examples of extracted ion currents. Indicated are representative examples of extracted ion currents from a peptide of Cdc42 and a peptide of IQGAP1 in each of the WT, CA, and DN conditions. Shown are the observed peaks from the monoisotopic precursor ion (blue), monoisotopic +one C13 ion (purple), and monoisotopic + two C13 ion (red). The Y axis represents relative intensity within each run, and X-axis corresponds to the retention time that each peptide eluted in each run. **B.** Quantification IQGAP1 Normalized to Cdc42. Extracted ion currents from 3 peptides of the bait protein, Cdc42, were used to normalize quantification of interacting proteins. Extracted Ion currents from three peptides of IQGAP1 were summed, normalized to Cdc42, and relative intensity was plotted. Error bars represent standard error of the mean, and asterisks represent significance of p<0.05.

Supplementary Movies: Real time imaging of 3D cellular invasion of spheroids embedded in matrigel using fluorescence imaging

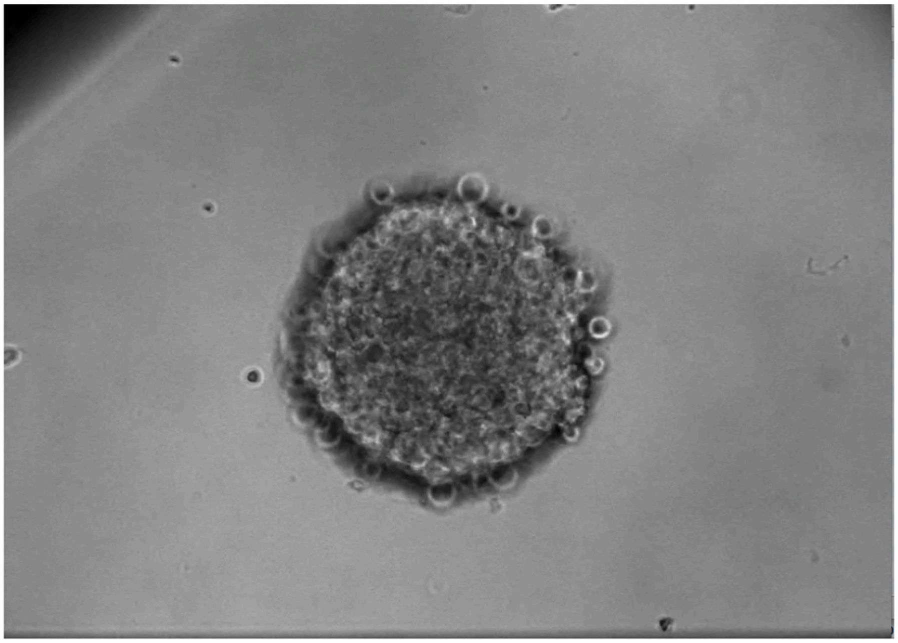

Supplementary Movies S1A: Uninduced control, spheroid clone does not express GFP (negative fluorescence) and is monitored by bright-field imaging.

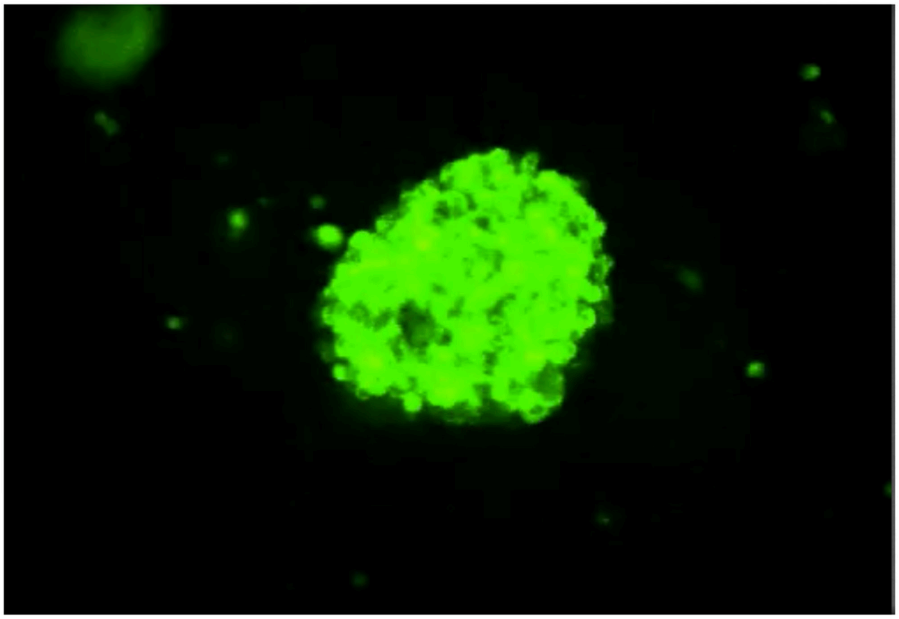

Supplementary Movie S1B: Active GFP:CA-Cdc42 expressing spheroids induced by doxycycline. Cells are rapidly invading compared to uninduced control.

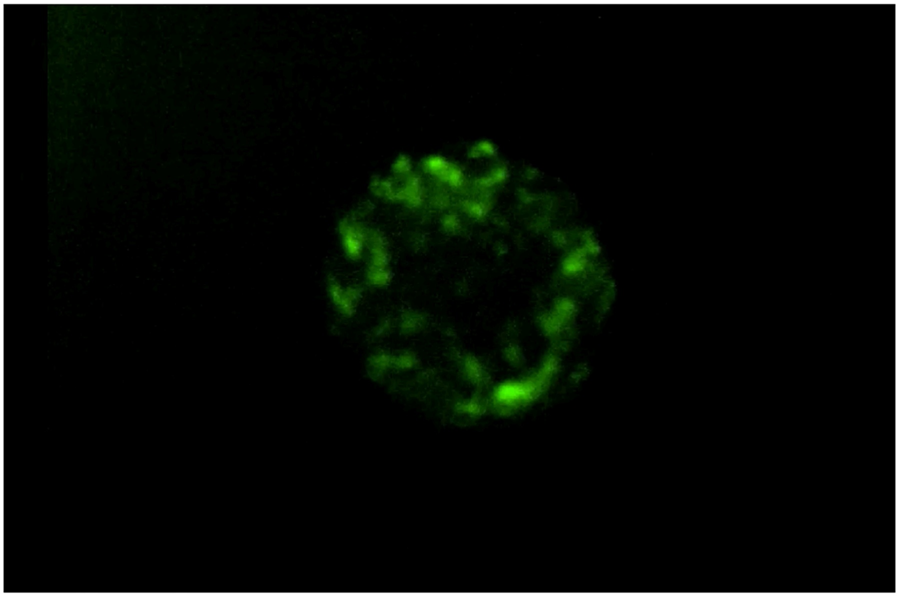

Supplementary Movie S1C: Inactive GFP:DN-Cdc42 expressing spheroids induced by doxycycline. Cells are slowly invading compared to uninduced control.
